# Population Vulnerability to the SARS‐CoV‐2 Virus Infection. A County‐Level Geographical‐Methodological Approach in Romania

**DOI:** 10.1029/2021GH000461

**Published:** 2021-11-01

**Authors:** Bianca Mitrică, Irena Mocanu, Ines Grigorescu, Monica Dumitraşcu, Adriana Pistol, Nicoleta Damian, Paul‐Răzvan Şerban

**Affiliations:** ^1^ Human Geography and Regional Development Department Institute of Geography Romanian Academy Bucharest Romania; ^2^ Environmental Geography and GIS Department Institute of Geography Romanian Academy Bucharest Romania; ^3^ Physical Geography Department Institute of Geography Romanian Academy Bucharest Romania; ^4^ National Institute of Public Health Bucharest Romania

**Keywords:** population vulnerability, SARS‐CoV‐2 virus infection/COVID‐19 disease, geographical‐methodological approach, NUTS3/County level, Romania

## Abstract

The assessment and identification of risk/vulnerable groups and risk factors are vital elements that can help quantify the pandemic potential of the SARS‐CoV‐2 virus in order to plan prevention and treatment measures. The aim of the study is to identify a methodological approach of population vulnerability to the SARS‐CoV‐2 virus infection. The study identifies reliable data sources and sets up a unitary database with statistical variables, quantitative and qualitative indicators with potential for being updated and improved. The analysis takes into account a number of variables/indicators (e.g., elderly persons, population without physician care, number of people suffering from cardiovascular diseases, number of people suffering from respiratory diseases, dwellings not connected to the public water supply network, no. of medical staff, number of COVID‐19 hospitals, PCR testing laboratories, number of vaccinated persons) grouped into the key vulnerability components: exposure, sensitivity, coping capacity and adaptive capacity. They allowed the computation of the final Index of Population Vulnerability to the SARS‐CoV‐2 virus infection and the mapping of different dimensions of vulnerability. The study was performed using the statistical data available at NUTS3/County level provided by different institutions (e.g., the Ministry of Health, the National Institute of Public Health, the Strategic Communication Group, and the National Institute of Statistics). The mapping of the different degrees of vulnerability could solve a problem of visibility for possible areas with vulnerable population, but also a problem of communication between different institutional health and administrative levels, as well as between all of them and the local communities and/or professionals.

## Key Points

- Risk/vulnerable groups and risk factors are vital elements that can help quantify the pandemic potential of the SARS‐CoV‐2 virus
- The mapping of the different degrees of vulnerability could solve a problem of visibility of possible areas with vulnerable population
- The results could lead to a rethinking of the interventions of the decision‐makers

## 1 Introduction

At international level, specialties such as health geography or epidemiology make an important contribution to explaining the spatial distribution of diseases (Jones & Moon, 1987; Khan, 1971; Laugier, 1902), to identifying and ranking risk factors at territorial level (e.g., Berke, 2010; Dumitrache, 2009; Marmot, 2005), according to which public health institutions plan their specific services and activity and develop health policies.

Vulnerability is a theoretical concept (Patt et al., 2008), and from the perspective of social sciences it can be a starting point in risk assessment and reduction, as it is considered an internal risk factor of a system before it faces a certain type of disruptive event; it represents the physical, economic, social susceptibility or tendency of a community to deteriorate in the event of threatening conditions, of natural or anthropogenic origin (Cardona, 2003; Emrich & Cutter, 2011). Generally speaking, vulnerability can be defined as the inability of a system to withstand the disturbances of external factors (Adger, 2006). The concept of vulnerability is frequently used in the public domain, in healthcare, social institutions and multidisciplinary research (de Groot et al., 2019) such as economics, demography, sociology, geography, anthropology, disaster management, environmental sciences, medicine etc.

Population vulnerability refers to the demographic categories likely to suffer from the perspective of physical, mental or precarious economic and social health. Theoretically, any person, at any given time, as a result of life circumstances, can be vulnerable (de Chesnay, 2015; Lee & Scanlon, 2007). The concept of vulnerable population refers to vulnerability in terms of the "status" that is, some demographic groups are vulnerable, at some point, in relation to other individuals or groups, by reference to any other type of extreme natural or man‐made event. There are several conceptual models for analyzing and measuring the population’s vulnerability to extreme events such as: (a) the “doubleˮ structure of vulnerability: an external component (risk exposure) and an internal one (adaptability and response), according to Bohle et al. (1994); (b) vulnerability as a component of risk (along with hazard, exposure, and adaptability), with four dimensions: physical, social, economic, and environmental (Bollin et al., 2003); (c) the holistic approach of risk and vulnerability; (d) the comprehensive model involves the analysis of the exposure, sensitivity, and adaptability components (Birkmann, 2006; Turner et al., 2003). This conceptual model is based on the idea that, in a territory, vulnerability is dependent on: (a) potential exposure — the degree to which the territorial system is exposed to significant changes or extreme events; (b) sensitivity — the measure with which the territorial system or a component changes in correlation with a stress factor, the size of which is determinable (Mac & Petrea, 2002); (d) coping capacity — the ability of the system to continue to operate (with modified parameters), even in the case of a hazardous event; (e) adaptive capacity — the ability of the system to adapt to changes, to reduce damages (Adger, 2006; Brooks et al., 2005; Turner et al., 2003). Each of these vulnerability components is expressed through statistical indicators so that the vulnerability can be ultimately assessed and differentiated according to degrees of intensity at the territorial level. The conceptual model of vulnerability used in international studies follows a bottom‐up approach (from local to regional and national scales) (Adger, 2006; Turner et al., 2003).

Generally, population vulnerability can be assessed in relation to different types of hazardous events (especially natural, i.e., climate‐related) and differentiated at various territorial levels (mainly national and regional), as reflected in recognized studies by Adger (2006); Adger et al. (2007); Brooks et al. (2005); Cutter (2003); Flanagan et al. (2011); Füssel (2010); Hinkel (2011); Turner et al. (2003).

Despite a number of pioneering studies trying to explain the role of some intrinsic factors, assessing the population's vulnerability in relation to pandemics is a relatively new direction in geographical literature. The novelty is not a limitation, but an opportunity to adapt to the research directions opened up by the new context of the SARS‐CoV‐2 virus. The elderly and those with pre‐existing chronic health conditions may be at high risk of developing severe health consequences from COVID‐19 (Bloom, 2020; Wyper et al., 2020). Vulnerability to disasters such as COVID‐19 is socially constructed (Burton et al., 2018) and highlights the social, economic, demographic, and geographic characteristics that shape not only the risk exposure, but also the capacity to deal with, respond to, and recover from disasters and hazards (Kim & Bostwick, 2020).

The occurrence of environmental disturbances, that is, the COVID‐19 pandemic, comes to accentuate some pre‐existing imbalances such as social inequities, racial/ethnic gaps, aging, illness etc. Wyper et al. (2020) consider the elderly population share and Years Lived with Disability (YLD) to highlight the populations that were most likely to be vulnerable to severe health consequences from COVID‐19, placing Romania among the top 10 countries with higher YLD rate for COVID‐19 vulnerable health conditions for the category 70 years and above. In support of these results are the studies conducted by DeCaprio et al. (2020) and Hägg et al. (2020) stating that age, frailty, and comorbidity were considered predictive factors for COVID‐19 effects. Other studies demonstrated that COVID‐19 affected differently communities subject to racial inequality and social exclusion (Kim & Bostwick, 2020), homelessness (Banerjee & Bhattacharya, 2020), low and middle income (Cénat, 2020), or poverty (Tavares & Betti, 2020).

In Romania, population health was approached from different perspectives in terms of the dynamics/analysis of health and risk factors at national, regional or local level, the geographical distribution of health resources, the access to health services by the population, health policies (Dumitrache, 2003, 2004, 2009, 2015; Dumitrache et al., 2016, 2020; Iftimoaei & Baciu, 2018; Pop, 2010; Vlădescu et al., 2016; Zamfir et al., 2015). Numerous national (National Report on Population Health, 2019; National Health Strategy 2014–2020) and international (OECD Country Health Profile, 2019; European Observatory on Health Systems and Policies) reports highlighted various aspects of population health in terms of dynamics or comparative perspective, health system deficiencies, impact of public health measures. However, the current situation is unprecedented. Since the first case of COVID‐19 was reported (February 26th, 2020), Romania has experienced a significant growth in the number of cases and death, reaching 1,053,629 cases and 27,971 deaths by the end of April 2021 (according to the Strategic Communication Group, Press release, April, 29, 2021). Ever since, some national‐level studies have tried to capture some immediate key aspects of the COVID‐19 pandemic in support of vulnerability evaluation: early spread and the first human‐to‐human transmission networks (Hâncean, Perc & Lerner, 2020), the potential risk of COVID‐19 to the health of people with disabilities in residential care institutions (Safta‐Zecheria, 2020), labor market vulnerabilities (Chivu & Georgescu, 2020), effects on the budgetary mechanism established to cover public health expenditure (Onofrei et al., 2021), impacts on society and the environment as perceived by the more highly educated population (Matei et al., 2021), challenges posed to the health of people with disabilities (Safta‐Zecheria, 2020). However, to understand the dynamics of the spread of the SARS‐CoV‐2 virus, complex studies are needed to consider, in addition to the social, economic, health and spatial dimension of the phenomenon, the identification of vulnerable territories and the exposed population. At national level, the studies addressing the territorial dimension of the SARS‐CoV‐2 virus are few and far between, given the novelty of the subject. One of the first studies was carried out by a research group from the Faculty of Sociology and Social Work (Bucharest University) and analyzed the first 18 days of the SARS‐CoV‐2 spread in Romania, highlighting the means of infection (contagion) through statistical modeling (Hâncean, Vică, et al., 2020).

The role that the elements of vulnerability of the population play in the spread of the COVID‐19 pandemic is a subject under‐analyzed in Romania. Thus, the present study aims (a) to complete the research already carried out, (b) to build a database at county level (with the possibility of synthesis at national level, in order for it to be used by different beneficiaries in various fields — healthcare units, territorial administrations etc.). The study also aims at (c) developing a methodology, and (d) identifying the different levels of population vulnerability to the SARS‐CoV‐2 virus infection and the territorial disparities at county level (NUTS3) through the empirical examination of their underlying factors (e.g., elderly persons, population without physician care, number of people suffering from cardiovascular diseases, number of people suffering from respiratory diseases, dwellings not connected to the public water supply network, no. of medical staff, number of COVID‐19 hospitals, PCR testing laboratories, number of vaccinated persons). Three different research directions were taken into account: (a) establishing the statistical variables and indicators for identifying the population vulnerability to the SARS‐CoV‐2 virus infection; (b) calculating secondary indices: the potential exposure (PE_SARS‐CoV‐2), population sensitivity (S_SARS‐CoV‐2), coping capacity (CC_SARS‐CoV‐2) and adaptive capacity (AC_SARS‐CoV‐2) of the population in the new pandemic context; (c) assessing the levels of population vulnerability based on the Population Intrinsic Vulnerability to the SARS‐CoV‐2 virus infection Index (PIV_SARS‐CoV‐2).

## 2 Study‐Area

**Figure 1:**
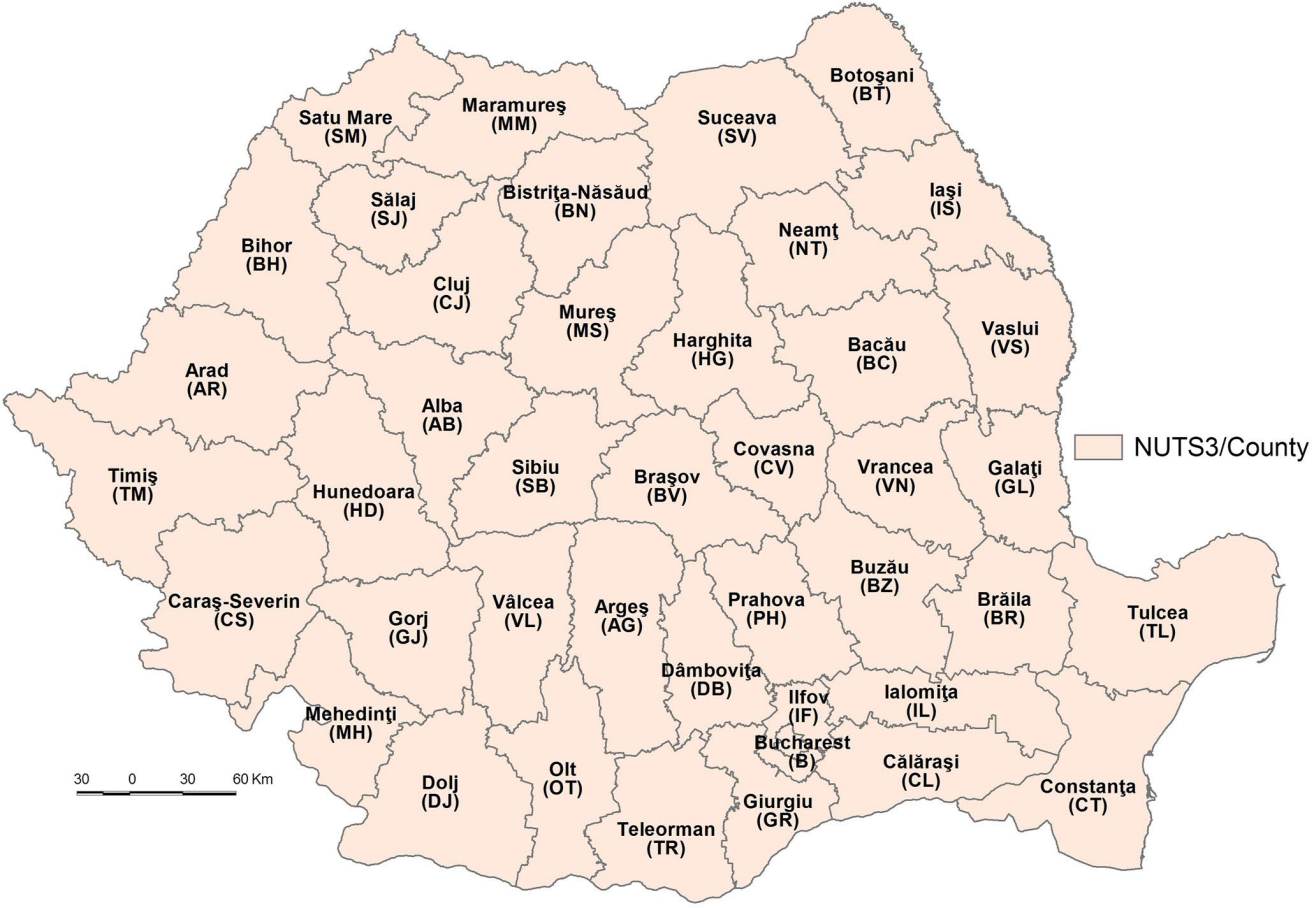
The territorial organization of Romania at the NUTS3 level.

The current analysis is performed at NUTS3/county level, which is the traditional administrative‐territorial unit level in Romania. The territory of Romania is organized in 41 counties and Bucharest Municipality, representing associations of various numbers of NUTS5/LAU, that is, a total of 319 urban LAUs (towns) and 2,862 rural LAUs (communes) Figure 1.

The geographical, historical, economic and socio‐political conditions influence the demographic size of the counties, a very important element for population exposure to the SARS‐CoV‐2 infection. The economic features have also an important role on healthcare and education system, sanitation facilities. The Government of Romania, as the central institution managing the COVID‐19 pandemic, has territorial representativeness at county level through its Ministries of Health (i.e., Prefectures, Public Health Directorates, the National Institute of Public Health), Internal Affairs (i.e., the Strategic Communication Group, the Romanian General Inspectorate for Emergency Situations, the Department for Emergency Situations), and National Defense.

## 3 Data and Methods

At the national level, significant results have been achieved in the territorial approach of population vulnerability to various external natural events: the results are numerous and can be used in the study of population vulnerability to the SARS‐COVID‐2 virus transmission through the adaptation of the indicators taken into account.

National‐ and regional‐level theoretical and practical research papers carried out, Armaş and Gavriş (2013); Dumitraşcu et al. (2018); Grigorescu et al. (2021); Mocanu et al. (2021); Stângă and Grozavu (2012); Tanislav et al. (2009) addressed population vulnerability to various extreme events (i.e., earthquake, flood, drought). The above mentioned methodologies and studies provide the background for the development of an index evaluating the population vulnerability to the SARS‐CoV‐2 virus infection. This approach entails a data base which should be comprehensive, but difficult to build, given that the COVID‐19 pandemic is a new experience for worldwide academic and institutional parties.

The data base and all the information referring to the COVID‐19 pandemic used and capitalized upon in this paper are built and collected up to the moment of “February, 2021” and for the county/NUTS3 territorial level. Despite the daily flows of information about the COVID‐19 pandemic in Romania, the official statistical data potentially reflecting the population’s vulnerability to SARS‐CoV‐2, and moreover, its territorial features, are limited. The official data sources used in this paper are the following: the Romanian Government/the Ministry of Health, the National Institute of Public Health (NIPH), the Strategic Communication Group, the National Committee for the Coordination of Vaccination Activities against COVID‐19, the National Appointment Platform for Vaccination against COVID‐19 (all of them under the coordination of the Ministry of Internal Affairs), the National Institute of Statistics (TEMPO‐Online database, http://statistici.insse.ro), the platforms https://stirioficiale.ro and https://datelazi.ro. Additionally, several official and unofficial data sources are used and capitalized upon in this study, since they use open‐source official information, updated daily: https://vaccinare-covid.gov.ro/platforma-programare/, https://covid19.geo-spatial.org/harti/hospital-infrastructure, and https://data.gov.ro/dataset/transparenta-covid.

Worldwide, the driving forces behind the COVID‐19 pandemic were explored through the exploitation of the experience gained by the international scientific community during and after the 2009 H1N1 pandemic (Borse et al., 2013; Kumar et al., 2012a, 2012b; Liu et al., 2011; Lowcock et al., 2012; O'Sullivan & Bourgoin, 2010; O'Sullivan et al., 2014; Tam at al., 2014; Wenger et al., 2011). Scientific reports on epidemics (Hay et al., 2013; O'Doherty et al., 2018) and especially on the current COVID‐19 pandemic (Wilder‐Smith et al., 2020; Williams et al., 2020) studied, adapted and applied complex information in order to calculate vulnerability indexes. Several papers and scientific reports made various indexes operational for epidemic risk assessment (e.g., Epidemic Risk Index, O'Doherty et al., 2018) or identified risk factors associated with the SARS‐CoV‐2 virus (Eurosurveillance Editorial Team, 2020; Smith & Judd, 2020; Sominsky et al., 2020; Williams, 2020).

In the context wherein the severity of infection and its rapid spread are overwhelming, computing the population vulnerability to the SARS‐CoV‐2 virus index constitutes both a priority, and a concern. Thus, DeCaprio et al. (2020) built the “CV19” index as an open‐source domain on the internet, with an emphasis on prioritizing and securing resources in time and space. The authors developed and computed two secondary indexes, namely the demographic vulnerability index (based on the % of people over 80 years of age, the % of people living in collective centers, such as old people, students etc., the % of multi‐generational households, the % of disabled population) and the vulnerability of the health status index (developed on the % of the population suffering from various ailments that are known or presumed to deteriorate the general health status in case of infection with the SARS‐CoV‐2 virus, the % of obese people, the % of smokers). Both indexes are expressed as the Z Score of the selected indicators. The researchers from Texas A & M College of Veterinary Medicine & Biomedical Sciences (2020) have developed an online tool for COVID‐19 risk assessment, namely, the Pandemic Vulnerability to COVID‐19 Index (PVI). The indicators selected reflect the virus transmission rate, population density, testing capacity, age group structure, comorbidity and health‐care infrastructure. Monitoring the vulnerable population at the regional level in the USA (COVID‐19 Healthcare Coalition, 2020) was based on the assumption that all communities are vulnerable to the community‐acquired SARS‐CoV‐2 virus and that vulnerability was based on three distinct but interrelated dimensions: the vulnerability of health status (or the medical vulnerability), the social vulnerability and the healthcare infrastructure vulnerability.

**Figure 2:**
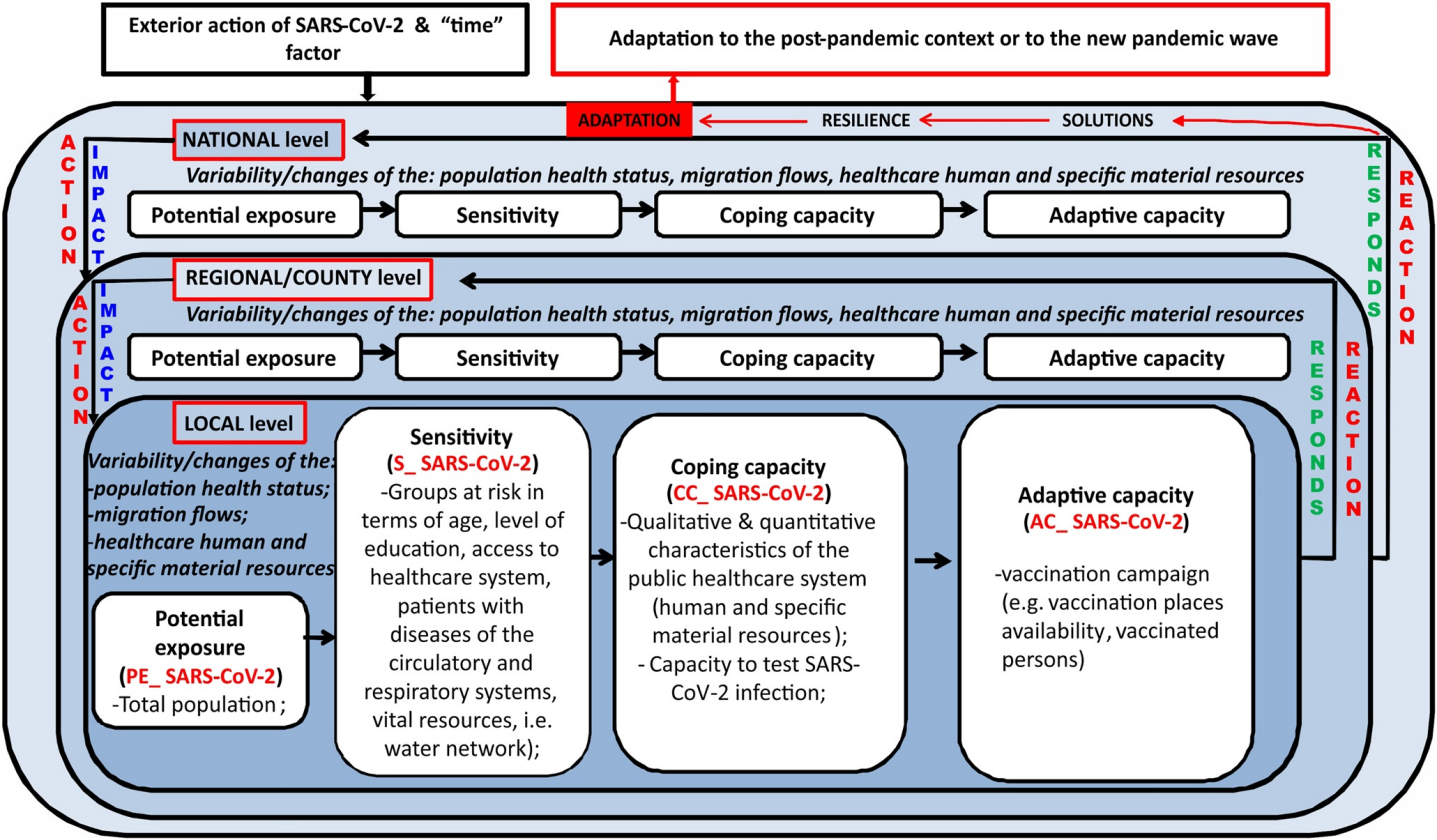
Conceptualization of the population intrinsic vulnerability to SARS‐CoV‐2 virus infection Index.

As mentioned above, there are a number of existing guidelines in literature to help build a suitable methodology able to assess the population’s vulnerability to the SARS‐CoV‐2 virus infection at national level. In view of that, the current paper follows the conceptual model based on the four vulnerability components (potential exposure, sensitivity, coping capacity and adaptive capacity, according to Adger, 2006; Brooks et al., 2005; Mac & Petrea, 2002, Turner et al., 2003), as shown in Figure 2 and Table 1.

**Table 1:** Population Vulnerability Indicators Selection

Vulnerability components/Secondary indexes	Variables & indicators selected	Description (i), data sources (ii) and measurement unit (iii)	Acronym	Influence
Potential Exposure PE_SARS‐CoV‐2	1. Population	(i) Number of persons with Romanian citizenship and permanent residence at LAU level; (ii) NIS, TEMPOnline database; (iii) number of persons	POP	+
Population sensitivity S_SARS‐CoV‐2	2. Access to physician care	(i) Share of the population without physician care of the total population of the county; (ii) NIS, TEMPOnline database; (iii) %	POP‐NO‐PHYS	+
	3.Vulnerable age group	(i) share of elderly persons (65 years old and over); (ii) NIS, TEMPOnline database; (iii) %	AGE	+
	4. People suffering from cardiovascular diseases (CVDs)	(i) number of people suffering from cardiovascular diseases; (ii) NIPH; (iii) no./100,000 inh.	CVDs	+
	5. People suffering from respiratory diseases (RDs)	(i) number of people suffering from respiratory diseases; (ii) NIPH; (iii) no./100,000 inh.	RDs	+
	6. Education	(i) primary and secondary school dropout rate; (ii) NIS, TEMPOnline database; (iii) %	LOW‐EDUC	+
	7. Drinking water network	(i) share of dwellings not connected to the public water network supply of total dwellings in the county; (ii) NIS, TEMPOnline database; (iii) %	NO‐WATER	+
Population coping capacity CC_SARS‐CoV‐2	8. Hospitals	(i) number of COVID‐19 hospitals (ii) Ministry of Health; (iii) no./100,000 inh.	HOSP‐COVID	–
	9. Assistance and Intensive Care	(i) no of Assistance and Intensive Care bed capacity (ii) Ministry of Health; (iii) beds/1,000 inh.	AIC	–
	10. PCR testing laboratories	(i) no. of PCR testing laboratories (ii) Ministry of Health; (iii) no./100,000 inh.	LAB‐PCR	−
	11. Healthcare staff	(i) no. of medical personnel (ii) NIS, TEMPOnline time series; (iii) no./1,000 inh.	MED‐PERS	−
Population adaptive capacity AC_SARS‐CoV‐2	12. Vaccination places availability	(i) available places in vaccination locations; (ii) Romanian Government; (iii) places/1,000 inh.	VACCIN‐PL	−
	13. Vaccinated persons	(i) number of vaccinated persons; (ii) NIPH; (iii) no./1,000 inh.	VACCIN‐PERS	−

The methodological approach follows several research stages: (a) Selecting the indicators (Table 1); (b) Normalizing the indicators; (c) Computing the indicators and the secondary indexes; (d) Computing the Population Intrinsic Vulnerability to SARS‐CoV‐2 virus infection Index (PIV_SARS‐CoV‐2); (e) Mapping the different dimensions of vulnerability and the final index of intrinsic vulnerability to SARS‐CoV‐2.

The validation of the selected indicators has been done using the expert judgment approach and the focus group technique (Dumitraşcu et al., 2018; Grigorescu et al., 2021; Mocanu et al., 2021): (a) an institutional focus group consisting of several institutions (e.g., the National Center for Disease Surveillance and Control, the Romanian General Inspectorate for Emergency Situations) and (b) an academic focus group of experts in medical geography, public health, spatial analysis, epidemiology and health management. The members of the two focus groups were asked to express their opinions in terms of selection, processing and interpretation of indicators which could significantly improve the methodology. Both meetings were held in Bucharest and were hosted by the National Institute of Public Health.

The normalized value of the statistical indicator “X” for the “i” county is X_n_ = X_i_/X_nat_average_, where X_i_ is the absolute value of the statistical indicator “X” for the county “i,” and X_nat_average_ is the national average value of the X_i_ indicator.

Each of the four components of the vulnerability mirrored by the secondary indexes and by the population’s intrinsic vulnerability to the SARS‐CoV‐2 virus infection index (PIV_SARS‐CoV‐2) were computed as a Hull Score with a mean of 50 and a standard deviation of 14 (Cohen & Holliday, 2001), the equations being as follows:

**PIV_SARS‐CoV‐2** = 50 + 14*(POP + POP‐NO‐PHYS + AGE + CVDs + RDs + LOW‐EDUC + NO‐WATER – HOSP‐COVID – AIC – LAB‐PCR – MED‐PERS – VACCIN‐PL – VACCIN‐PERS)/13

We note that the indicators with a direct effect on PIV_SARS‐CoV‐2 have been deemed positive (“+,” e.g., the share of population aged 65 and over of the total population), and those with a reverse effect were assumed to be negative (“−,” e.g., the locations available for vaccination/1,000 inh.). The abilities of a socio‐economic system to continue functioning (namely, its coping capacity) by adapting to change (namely, its adaptive capacity) lower the degree of vulnerability. In view of that, we would like to mention that mapping the CC_SARS‐CoV‐2 and AC_SARS‐CoV‐2 secondary indexes involves a reverse representation, namely a reverse color scale. This is an adequate way of picturing them more correctly, in terms of its intrinsic significance, and realistically, in terms of county territorial level.

The values registered by the final index allowed for the classification of the degrees of the population’s vulnerability to the SARS‐CoV‐2 virus and of mapping that at the level of each county. The scaling of vulnerability to the SARS‐CoV‐2 virus is done according to the results obtained from the PIV SARS‐CoV‐2 vulnerability index score ranging from 47.520 (least vulnerable) to 52.650 (most vulnerable).

The approach of the methodological study on population vulnerability to the SARS‐CoV‐2 virus infection presents a series of (a) uncertainties and (b) limitations.

a The main ***uncertainties*** are the result of:
1. The lack of a centralized inventory, which is now organized in various forms of storage and is accessed by many users or beneficiaries;
2. The lack of existing data in digital format, which would allow for the creation of representative spatial database;
3. The lack of a typological harmonization that would enable the achievement of representative inventories at local, regional or national level (i.e., in the case of vulnerable groups, of vaccinated persons);
4. The fragmented data collection system related to healthcare activity, staff, and infrastructure.
b The ***limitations*** resulted from:
1. The general connections that have been made between the state of the population’s health, its level of socio‐economic development, and healthcare staff and infrastructure. These relations that were made only at a very general level haven't permitted the establishment of some spread patterns of SARS‐CoV‐2 virus infection;
2. If the variables related to exposure and sensitivity are relatively constant, those related to coping capacity and adaptive capacity are very dynamic; It is the case of indicators such as vaccination locations and vaccinated persons which have significantly increased as a result of the reinforcement of vaccination campaigns (i.e., “Vaccination Marathon” between May 7 and 10, when citizens and foreign residents could get the Pfizer shot without prior appointment), the increase in the number and diversification of vaccination centers (including malls and drive‐thru) and the variety of vaccine options (Pfizer‐BioNTech, Moderna, Oxford/AstraZeneca, Johnson & Johnson).

## 4 Results

**Figure 3:**
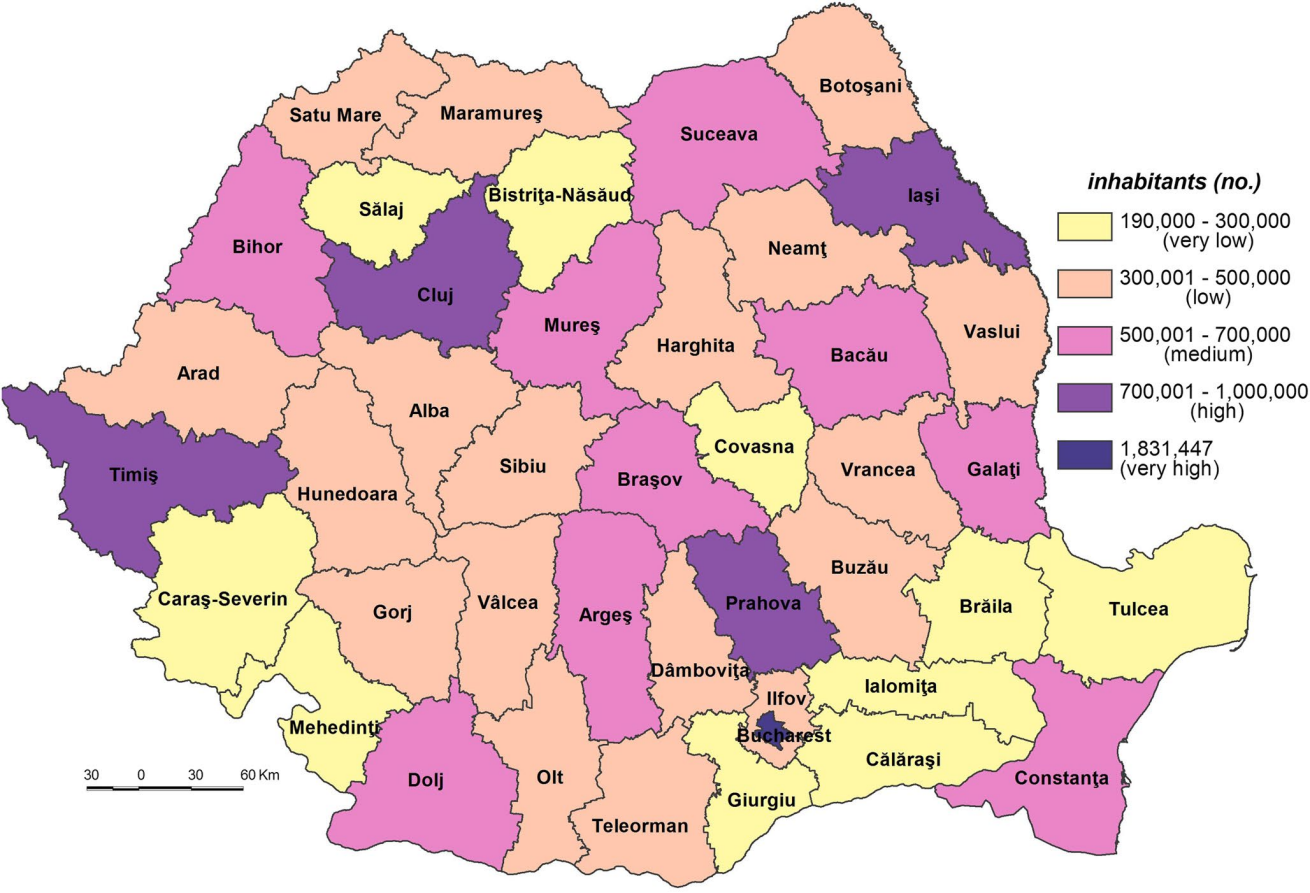
Potential exposure to the SARS‐CoV‐2 virus infection (PE_SARS‐CoV‐2).

The **Potential Exposure to the SARS‐CoV‐2 virus infection (PE_SARS‐CoV‐2)** Index is based on the population’s intrinsic characteristics with a potential of bringing to light its exposure to this virus infection. Because of their demographic amplitude, Bucharest (the Capital city) together with other counties where the largest Romanian cities are located are potentially exposed to the SARS‐CoV‐2 virus infection. Thus, the very high exposure was typical for Bucharest Municipality, which has a population of 1.8 million inhabitants, the demographic potential exceeding twofold the county next in line. High exposure was also registered by Iaşi, Prahova, Cluj and Timiş Counties, with a population ranging between 792,720 and 705,534 inhabitants (Figure 3). The counties of Iaşi, Cluj and Timiş owe their population size to the big cities of Iaşi, Cluj‐Napoca and Timişoara, which are their respective county‐seats, the next in line within the demographic hierarchy after Bucharest, the growth poles with services and universitary, cultural and research functions. The Prahova County displays a high degree of industrialization and urbanization. In the class of medium exposure nine counties are included, having different features that account for their demographic potential. Constanţa County owes its demographic‐size to the presence of the international harbor and to the growth pole ‐ Constanţa city. Dolj County is marked by the presence of Craiova growth pole, with diversified economic activities and a university center. The counties of Bacău, Argeş, Bihor, Braşov, Mureş and Galaţi, as industrialized counties, have a high influence in attracting demographic potential.

The low demographic potential varies from 301,983 inh. in Harghita County to 489,211 inh. in Dâmboviţa County. In this class 18 counties with different features are included: industrialized (Hunedoara, Gorj, and Vâlcea), ruralized (Teleorman, Olt, Dâmboviţa, Vaslui, Neamţ, and Botoşani), economically diversified (Sibiu and Arad), or agricultural and industrial (Satu Mare, Maramureş, Buzău, and Vrancea). The very low exposure is indicative of various counties from the South‐Eastern and Southern parts of Romania (Tulcea, Ialomiţa, Giurgiu, Călăraşi, and Brăila), and from the South‐Western (Mehedinţi and Caraş‐Severin) counties with a high degree of ruralization.

The **Population Sensitivity to the SARS‐CoV‐2 virus infection (S_SARS‐CoV‐2)**, meaning the degree to which the system is sensitive to or might be affected by the potential exposure, varies from a minimum value of 52.255 in Ilfov County to a maximum of 59.081 in Teleorman County.

**Figure 4:**
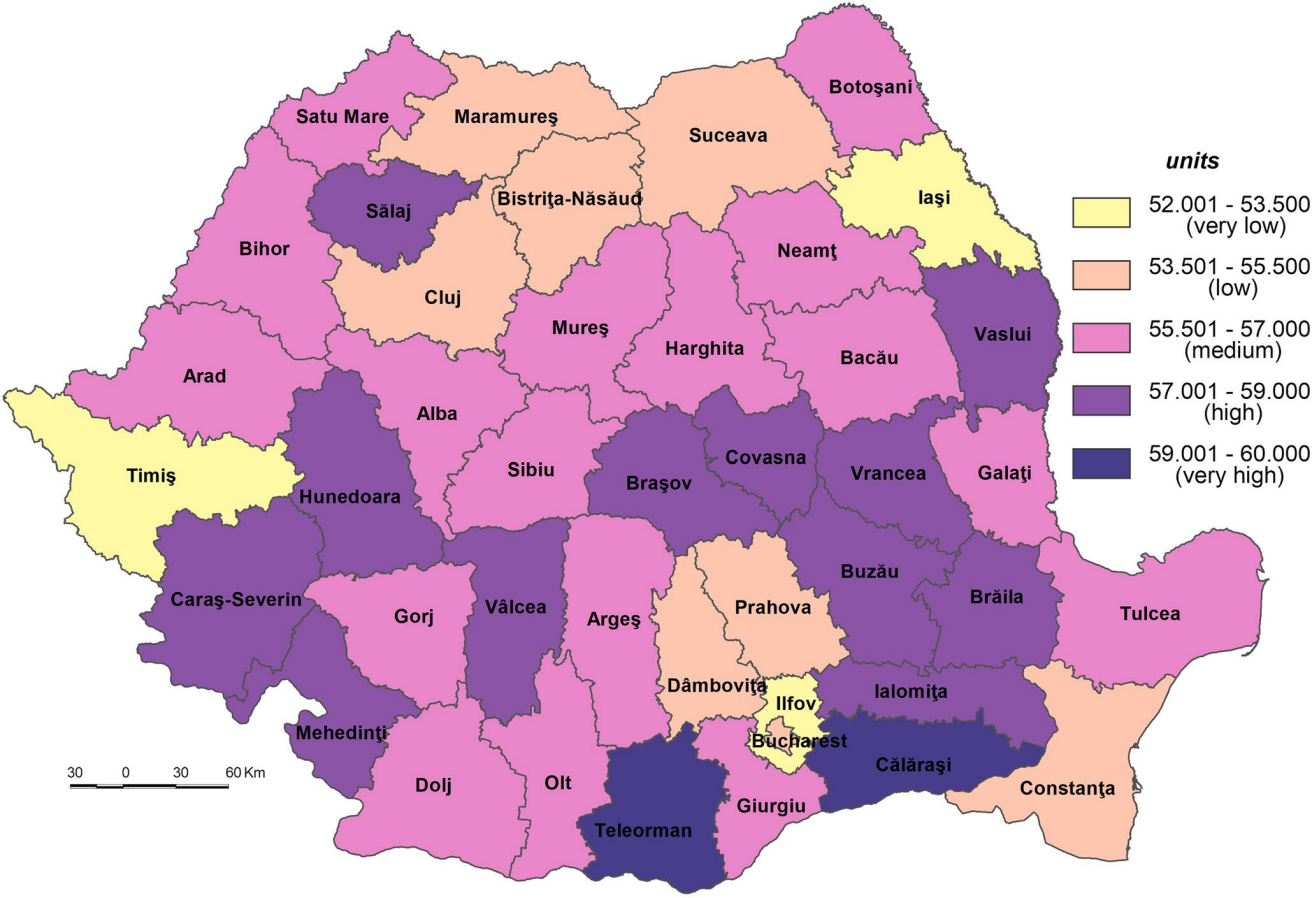
Population Sensitivity to the SARS‐CoV‐2 virus infection (S_SARS‐CoV‐2).

**Figure 5:**
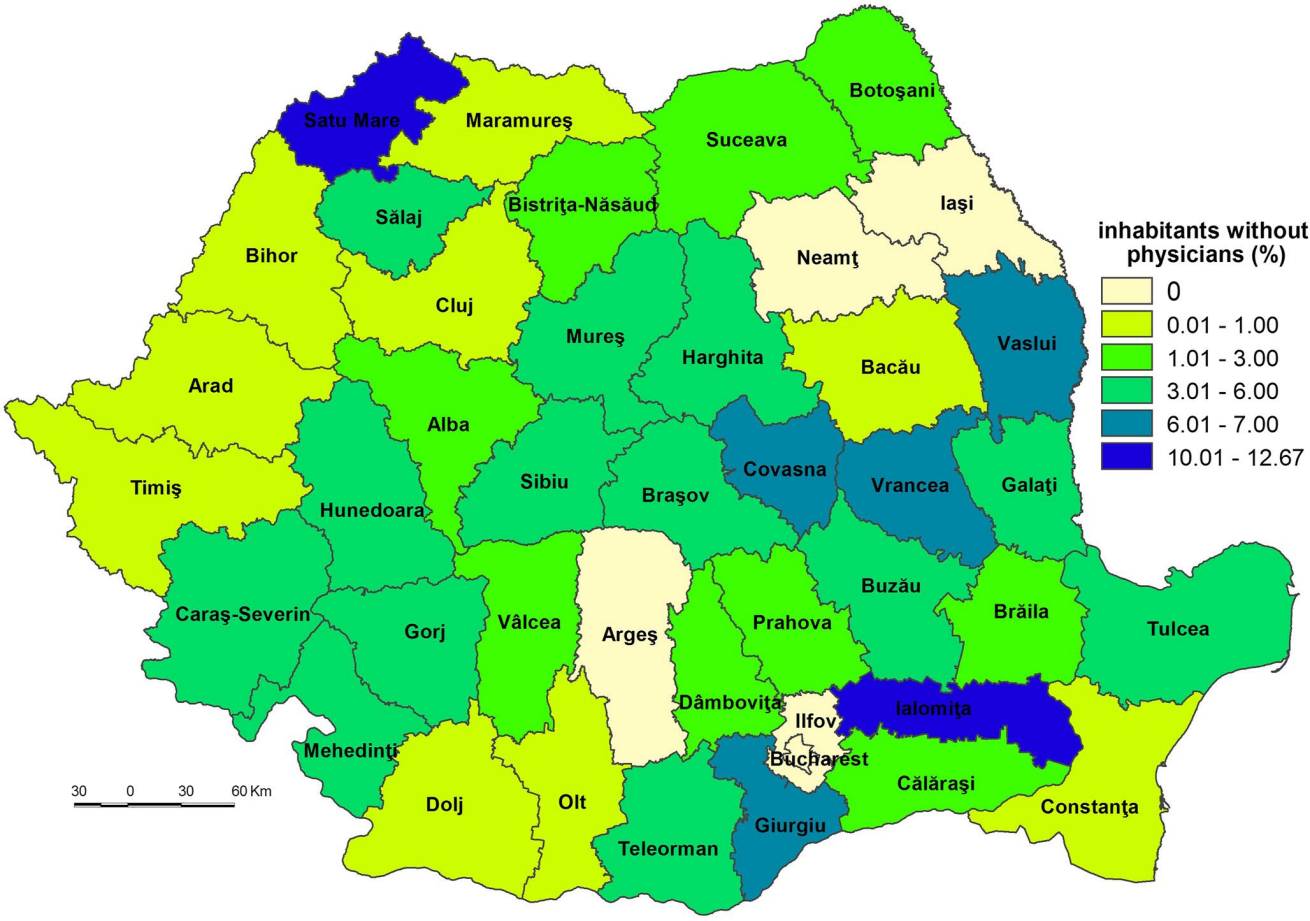
Population without access to physician care.

**Figure 6:**
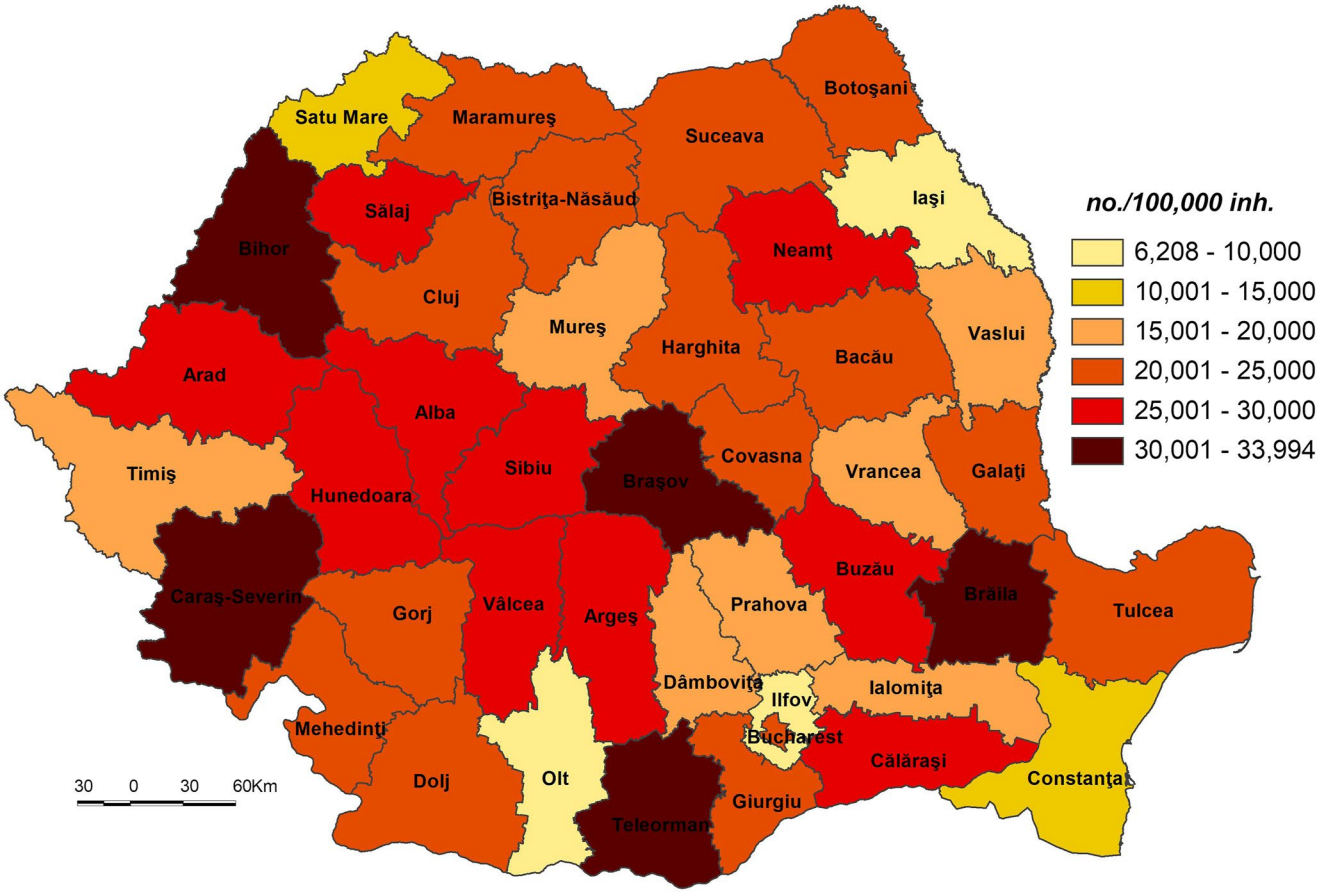
People suffering from cardiovascular diseases.

Apart from Ilfov County, Timiş and Ia'i Counties are included in the very low sensitivity class (52.001–53.500) (Figure 4). These low values are mainly due to the fact that the category of population without physician care doesn’t exist (in Ilfov County) or it is little represented (e.g., only 0.00% in Ia'i and 0.57% in Timiş, which are counties where the Romanian healthcare system has a higher‐than‐average performance (Figure 5)). The geographic location of medical services creates significant disparities in terms of healthcare access, which are subsequently reflected in the population health outcomes (Dumitrache et al., 2020). Cluj displays the lowest level of primary and secondary school dropout rate (0.8%), among the lowest share of dwellings not connected to the public water supply network (19.2%, respectively) and of elderly persons (24.5%). Ilfov and Ia'i County registers the lowest number of elderly persons (16.6%, respectively 22.8%), of number of people suffering from cardiovascular diseases (8,615 cases/100,000 inh., respectively 6,208 cases) (Figure 6), and of number of people suffering from respiratory diseases (891 cases/100,000 inh., respectively 700 cases).

**Figure 7:**
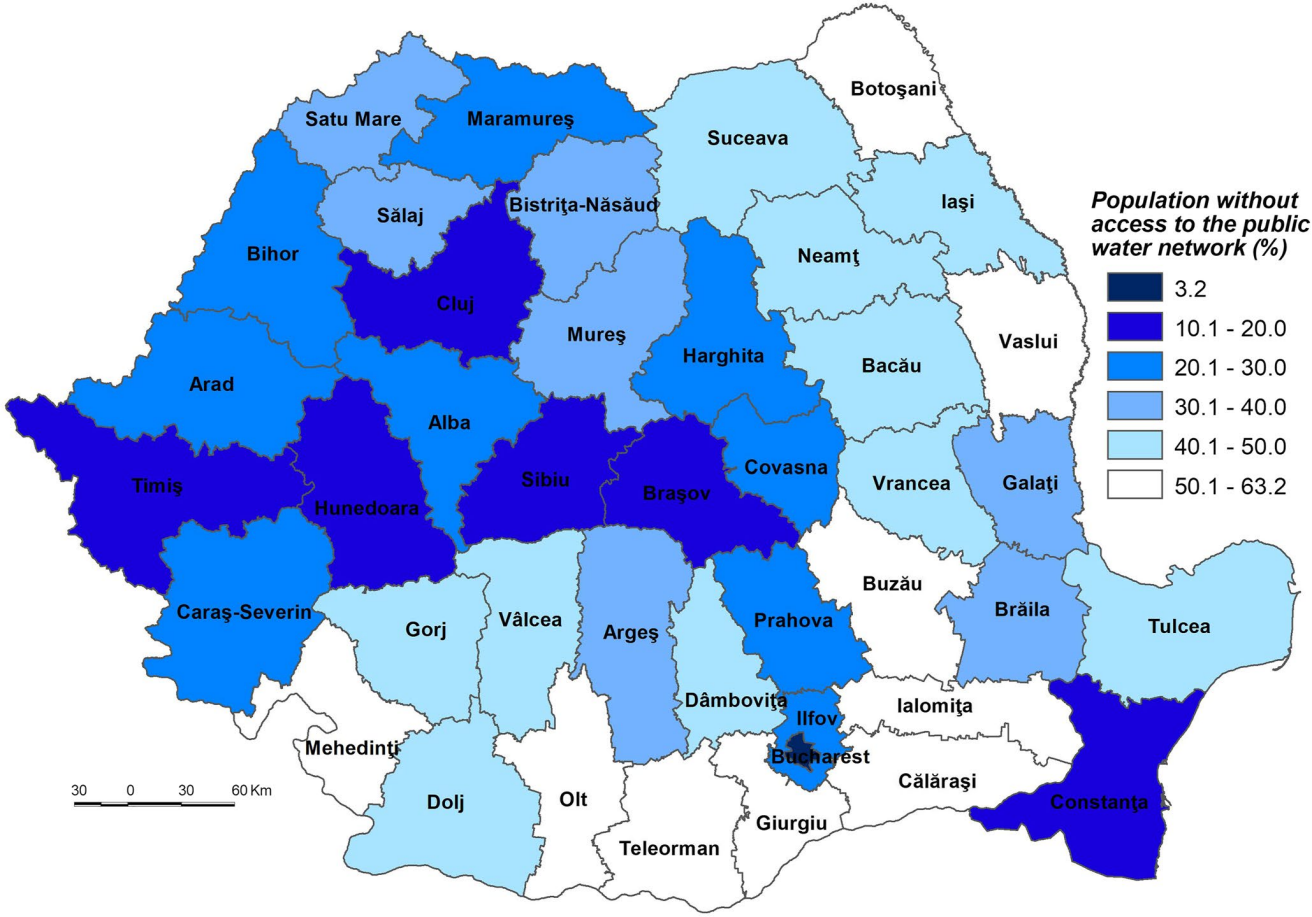
Population without access to the public water supply network.

**Figure 8:**
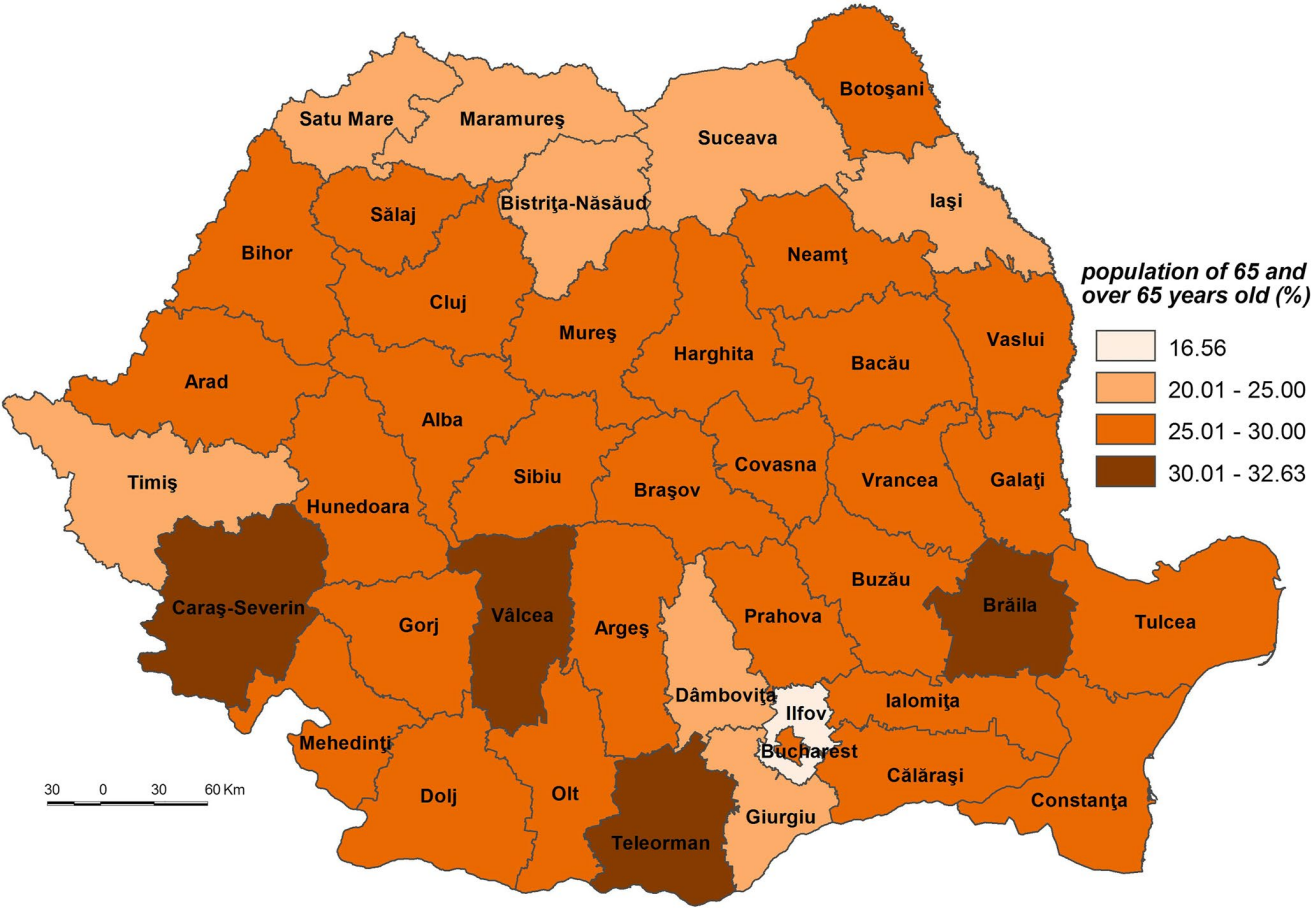
Population aged 65 and over.

The low sensitivity (53.501–55.500) is reflected by 7 counties (Constanța, Cluj, Prahova, Maramureș, Dâmbovița, Bistrița‐Năsăud and Suceava) and Bucharest Municipality. This level is due to the following indicators: (a) the share of the population without physician care of the total population which registers 0.00% in Bucharest Municipality, 0.37% in Cluj and 0.69% in Constanța (Figure 5), which are among the counties with a developed healthcare system (Dumitrache et al., 2020); (b) primary and secondary school dropout rate which registers the lowest values in Suceava (less than 1%), Bistriţa‐Năsăud, Dâmboviţa, Prahova, Bucharest Municipality and Maramureş (less than 1.5%); (c) the share of dwellings not connected to the public water supply network of total dwellings registers some of the lowest values in Bucharest Municipality (3.2%), Constanţa (15.3%) and Cluj (19.2%). These counties have a large number of town dwellers, generally connected to the water supply network (Figure 7); (d) number of people suffering from cardiovascular diseases is low in Constanța (13,457 cases/100,000 inh.), Prahova (16,323 cases), and Dâmbovița (18,271 cases); (e) number of people suffering from respiratory diseases is low in Constanța (1,281 cases/100,000 inh.), Prahova (1,771 cases) and Dâmbovița (2,221 cases); (f) in contrast to the previous indicators, the share of elderly persons is high in Bucharest Municipality, Prahova and Constanţa counties, ranging from 28.9% to 26.0%. However, this indicator does not have an even coverage at county level (Figure 8).

The medium sensitivity (55.501–57.000) includes 17 counties located in the central (Harghita, Alba, Sibiu, and Mureş), north‐eastern (Bacău, Neamţ, and Botoşani), south‐eastern (Galaţi and Tulcea), south‐western (Gorj, Dolj, and Olt), southern (Argeș and Giurgiu), north‐western (Satu Mare) and western (Arad and Bihor) parts of Romania (Figure 4). These counties have generally medium values, save for Olt, Botoşani and Giurgiu (high share of dwellings not connected to the public water supply network), Neamț and Tulcea (high share of elderly persons), Satu Mare, Giurgiu (high share of population without physician care), Dolj (high primary and secondary school dropout rate), Bihor, Sibiu, and Argeș (high number of people suffering from cardiovascular diseases) and Bihor and Argeș (high number of people suffering from respiratory diseases) which increases the degree of sensitivity. On the other hand, there are counties, such as Argeș, Neamț or Arad, with a small share of the population lacking physician care, or Gorj and Argeș–which has a low school dropout rate, Sibiu–with a small share of dwellings not connected to the public water supply network, Olt and Satu Mare with a low number of people suffering from cardiovascular diseases and Satu Mare and Galați with a low number of people suffering from respiratory diseases in which these indicators lower the degree of sensitivity.

The high sensitivity (57.001–59.000) is characteristic of 12 counties (Buzău, Ialomița, Covasna, Hunedoara, Mehedinți, Caraș‐Severin, Brăila, Bra'ov, Vrancea, Vâlcea, Vaslui, and Sălaj) (Figure 4). These areas are among the deeply socially disadvantaged in Romania located mainly in the southern part of the country, the majority of them well known for the very low degree of socio‐economic development, territorially concentrated in several counties–Vaslui, Vrancea, Brăila, and Mehedinți (Mitrică et al., 2020). This cluster of counties has among the highest shares of population without physician care and of dwellings not connected to the public water supply network, share of elderly persons. Covasna County registers the highest values of early school dropout (3.8%), Vaslui that of dwellings not connected to the public water supply network (63.2%) (Figure 7), Ialomița that of population without physician care (12.67%) (Figure 5). Bra'ov, Caraș‐Severin and Brăila have ones of the highest cases of people suffering from cardiovascular diseases (Figure 6) and Hunedoara, Sălaj, Brăila and Mehedinți of people suffering from respiratory diseases.

The very high sensitivity (59.001–60.000) is attributed to the counties of Teleorman and Călăraşi lying in the south of Romania (Figure 4). The highest share of early leavers reported in Romania is in rural areas (EUROSTAT, 2020), Călăra'i County, characterized by a second value of this indicator, having a high ruralization degree. In the same time, Teleorman has the highest share of elderly population (32.6%), highest number of people suffering from cardiovascular diseases and Călăra'i one of the highest values of people suffering from respiratory diseases. The level of connectivity of dwellings to the water network supply is under 50%.

**The Population’s Coping Capacity to the SARS‐CoV‐2 virus infection (CC_SARS‐CoV‐2)** is the population’s ability to respond to and recover from the effects of stress or disturbances caused by the infection with SARS‐CoV‐2 that have the potential to alter the structure or function of society (Bobrowsky, 2013). The CC_SARS‐CoV‐2 values vary from a minimum of 50.781 in Ilfov County to a maximum of 60.647 in Bucharest Municipality.

**Figure 9:**
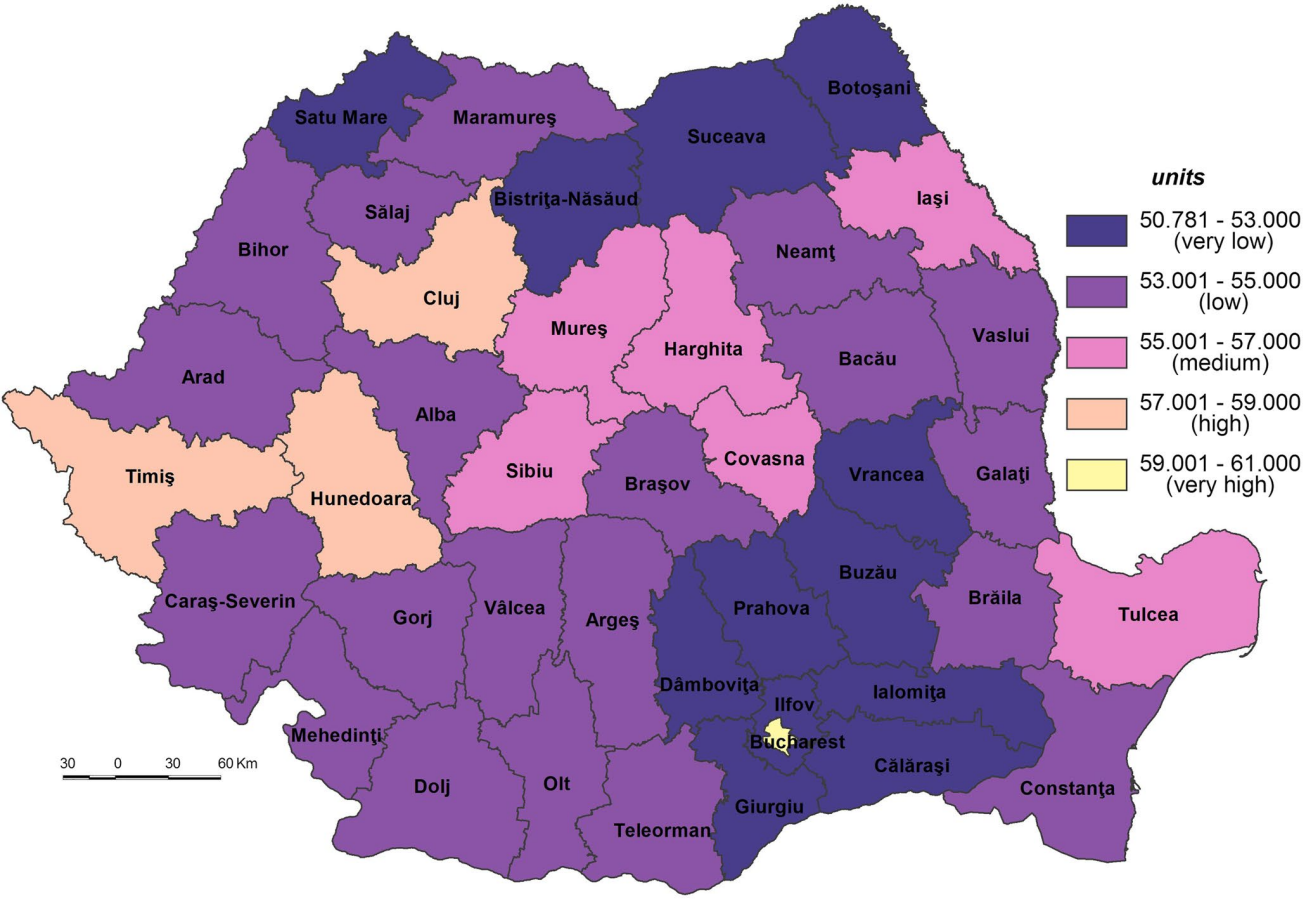
Population Coping Capacity to the SARS‐CoV‐2 virus infection (CC_SARS‐CoV‐2).

**Figure 10:**
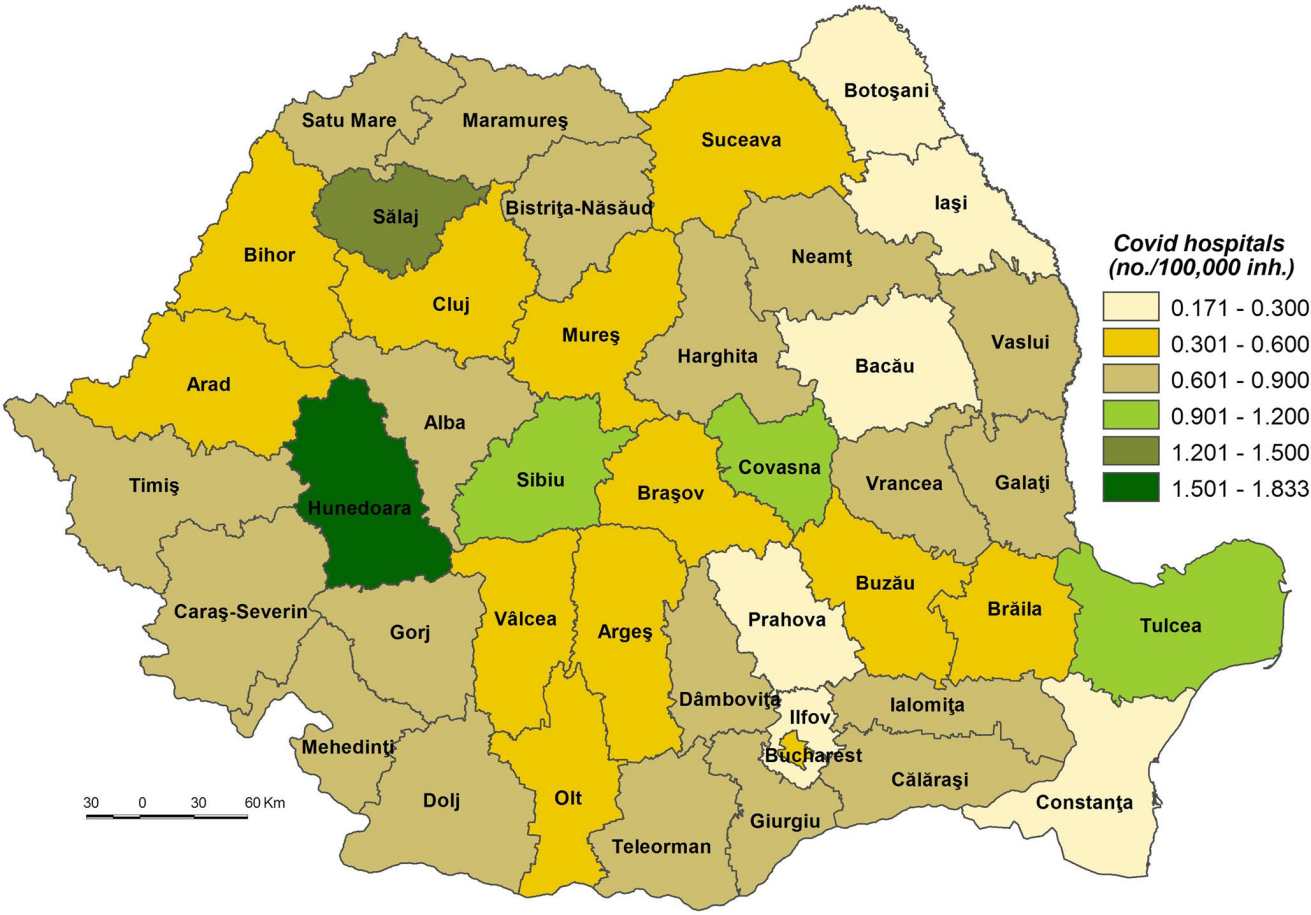
COVID‐dedicated hospitals.

The very low coping capacity (less than 53.000) is registered in Ilfov, Botoşani, Satu Mare, Giurgiu, Dâmboviţa, Ialomiţa, Prahova, Bistriţa‐Năsăud, Suceava, Buzău, Vrancea, and Călăraşi Counties (Figure 9). The low level of coping capacity is determined mainly by the low number of COVID‐19 hospitals reported in correlation with the number of inhabitants (Figure 10). For example, in Ilfov County there is no such hospital, while in Botoşani County there is only one. These types of hospitals have a special purpose and have been set up in order to treat COVID‐19 cases and support hospitals to minimize risks and ensure a separation of the flows of SARS‐CoV‐2‐positive patients from those who have not contracted this type of virus. Thus, in May 2020, 231 hospitals were designated to fight against SARS‐CoV‐2. Support hospitals are municipal health units, mainly with infectious disease departments, as well as hospitals from other networks: the Ministry of Transport, the Ministry of National Defense, private health units (Moldovan et al., 2020). The number of medical personnel is also at a low level, having the lowest values in Ilfov (1.94/1,000 inh.), Giurgiu (3.15), Ialomiţa (4.02), and Dâmboviţa (4.20). The Assistance and Intensive Care (AIC) has a vital role for patients that find themselves in a serious condition because of the SARS‐CoV‐2 virus infection. Given this, Ilfov, Giurgiu, Satu Mare, Dâmboviţa, and Ialomiţa Counties have the lowest values at the national level, between 0.04 and 0.11 AIC beds/1,000 inh. The testing capacity also plays an important role in detecting the SARS‐CoV‐2 infection, as all counties have values below the national average (0.9 PCR laboratories/100,000 inh), the lowest one being registered by the counties of Botoşani (0.26), and Satu Mare (0.30). Even Prahova County has a medium‐high level of socio‐economic development; its status is guaranteed by the high number of inhabitants assigned to the COVID‐19 hospital.

**Figure 11:**
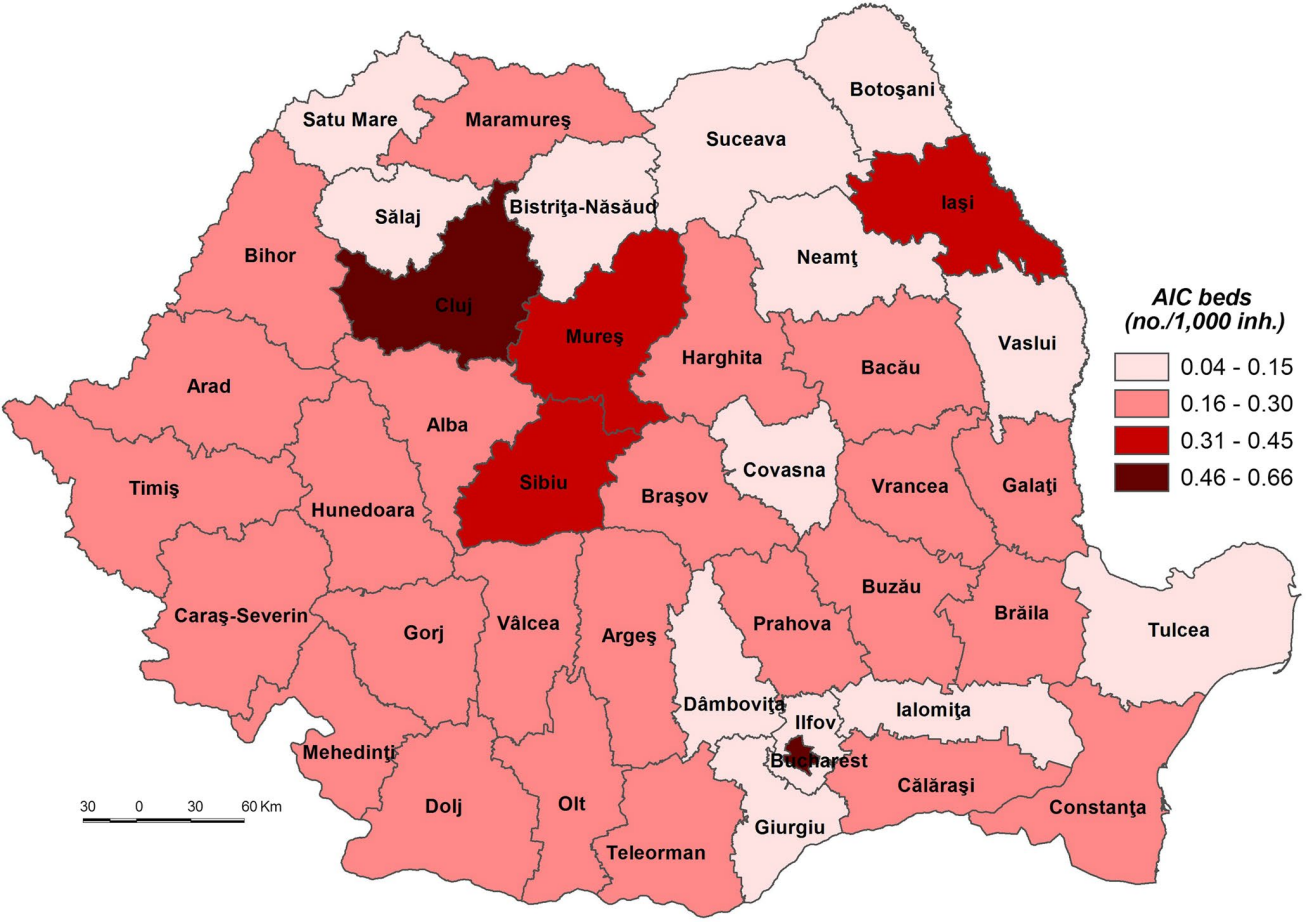
Assistance and intensive care bed capacity.

**Figure 12:**
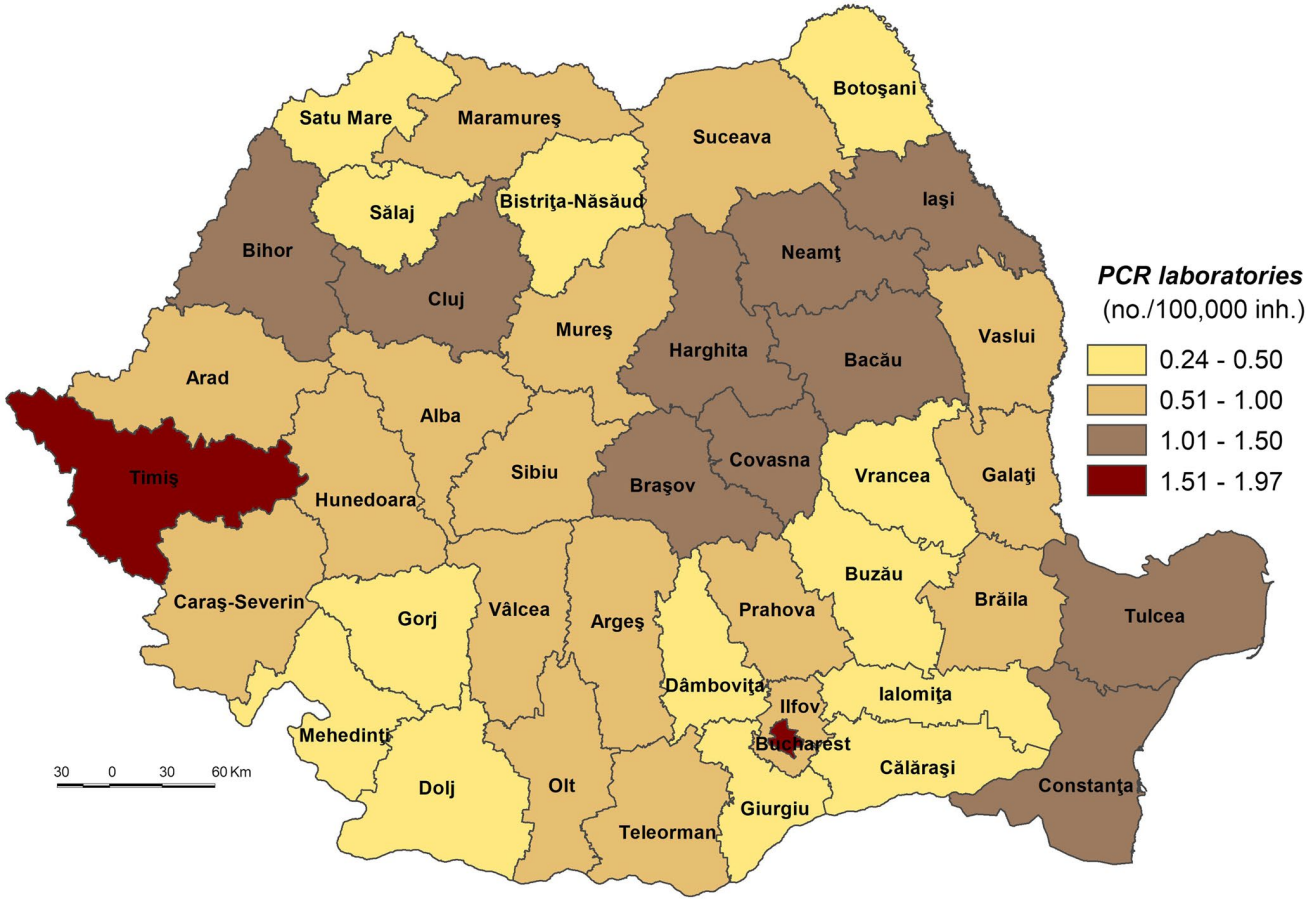
PCR laboratories.

The low coping capacity (53.001–55.000) comprises 20 counties, almost half of the total number of counties (Figure 9). This is due to the: (a) number of to COVID‐19 hospitals which varies from 0.17 hospitals/100,000 inh. in Bacău County to 1.83 hospitals/100,000 inh. in Hunedoara County in relation to the large number of COVID‐19 hospitals (7), with high values (over 0.70 COVID‐19 hospitals/100,000 inh.) included in Sălaj, Mehedinţi, Vaslui, Galaţi and Caraş‐Severin Counties and low values (less than 0.3) in Constanţa County (Figure 10); (b) Assistance and Intensive Care bed capacity ranging from 0.12 beds/1,000 inh. in Neamţ County to 0.30 in Caraş‐Severin County, the last being the only one with values over the national average (Figure 11); (c) the number of PCR testing laboratories below the national average (0.9) in 13 counties, Gorj County having 0.32 PCR testing laboratories/100,000 inh. and Braşov County — 1.27 (Figure 12); (d) the number of medical personnel varying from 4.36/1,000. to 8.16/1,000 inh. in Arad County and Dolj County, respectively.

The medium coping capacity (55.001–57.000) is reflected in six counties — Tulcea, Harghita, Mureş, Covasna, Sibiu and Iaşi — four of them located in the Central part of Romania (Figure 9). Tulcea, Sibiu and Covasna Counties are marked by a high number of hospitals per 100,000 inhabitants (between 1.035 and 0.991), while Iaşi County exhibits one of the lowest values, mainly due to the big demographic potential assigned to COVID‐dedicated hospitals (Figure 10). In terms of Assistance and Intensive Care bed capacity, Iaşi and Mureş Counties rank higher with values between 0.41 and 0.31 beds/1,000 inh (Figure 11). Additionally, in terms of PCR testing laboratories, Covasna, Harghita, Iaşi, Tulcea and Sibiu are defined by above‐average values (0.9 PCR laboratoires/1,000) (Figure 12). All six counties display values over the national average in terms of medical personnel, with Mureş and Iaşi ranking second and third.

The high coping capacity (57.001–59.000) is registered by Hunedoara, Cluj and Timiş Counties (Figure 9). Hunedoara ranks first in terms of COVID‐19 hospitals (1.833 hospitals/100,000 inh.), while Cluj and Timiş counties rank second in relation to the Assistance and Intensive Care bed capacity (0.51 beds/1,000 inh.) and PCR testing laboratories (1.56 laboratories/100,000 inh.).

Bucharest Municipality falls into the very high coping capacity class with a value of 60.912. The Capital‐city ranks first when it comes to medical personnel (12.99/1,000 inh.), Assistance and Intensive Care beds (0.66 beds/1,000 inh.) and PCR testing laboratories (1.97 laboratories/100,000 inh.).

The **Population’s Adaptive Capacity to the SARS‐CoV‐2 virus infection (AC_SARS‐CoV‐2)** comprises the properties of a system that enables it to modify itself in order to maintain or achieve a desired state in the face of perceived or actual stress (Jakku & Lynam, 2010). This aspect of vulnerability is assessed according to the locations available for vaccination (as of February 15, 2021) and the number of fully vaccinated persons (between December 2020 and February 2021). Both indicators reflect the concern for the development of adaptive capacity to the SARS‐CoV‐2 virus infection through an efficient and safe vaccine against the virus according to the EU Strategy on vaccines against COVID‐19 (https://ec.europa.eu/info/live-work-travel-eu/coronavirus-response/public-health/coronavirus-vaccines-strategy_ro) and, at national level, to the Strategy on vaccines against COVID‐19 (Decision no. 1,031/November, 27, 2020, http://legislatie.just.ro/Public/DetaliiDocumentAfis/234095). These two indicators reveal the relations between the total population of a county (as the “recipient” or beneficiary of the vaccination campaign) and the vaccination centers (and places), as a tool for implementing this campaign at territorial level, on one hand and, on the other hand, the practical result of the vaccination campaign set in motion with the purpose of aiding the population during the SARS‐CoV‐2 pandemic.

**Figure 13:**
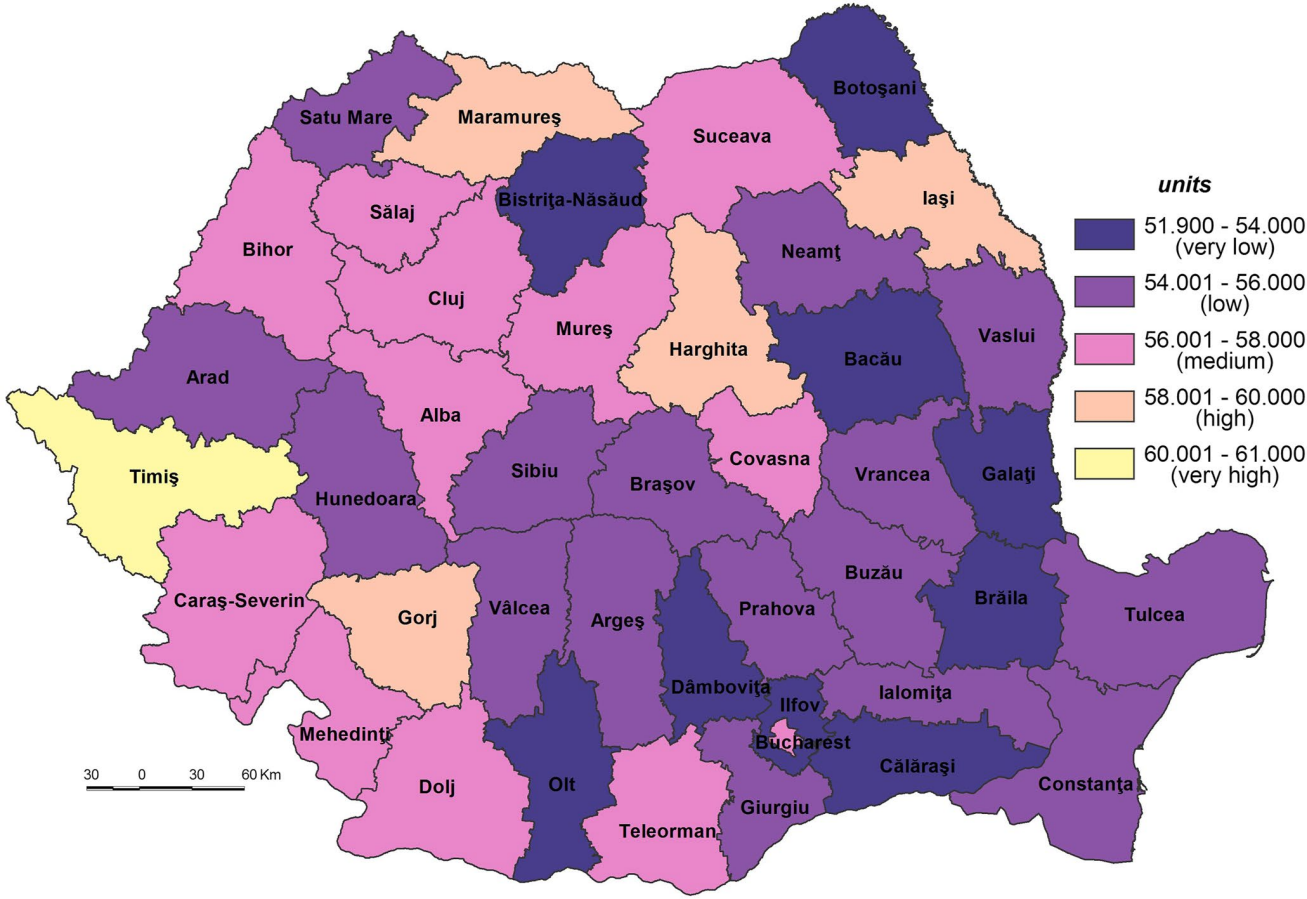
The population’s Adaptive Capacity to the SARS‐CoV‐2 virus infection (AC_SARS‐CoV‐2).

The AC SARS‐CoV‐2 values vary from a minimum of 49.998 in Ilfov County to a maximum of 63.996 in Timiș County (Figure 13). The lowest number of available places in vaccination locations/1,000 inhabitants is registered in Bucharest, Constanța and Cluj Counties, because of their large demographic size. The highest values of this indicator are recorded in Covasna and Harghita, both having 11.6 available places/1,000 inhabitants. The two counties have a small total population who benefit from a total number of 5,859 available places in vaccination locations distributed in 17 vaccination locations. Likewise, higher values can be found in Timiș (12.7 available places in vaccination locations/1,000 inhabitants), Gorj (10.9) and Teleorman (10.1) Counties and are impacted by several causes, such as the large number of vaccination centers (between 9 in Gorj and 12 in Timiș) and vaccination locations (i.e., Timiș has almost 9,000 available places in vaccination locations and the other two counties have 3,400, in comparison with the national average of 2,600 available places/county). Compare to the national average which was at the time about 5.7 available places in vaccination locations/1,000 inhabitants, 22 counties registered values over the average and 20 counties less than average. In almost half of the counties the values are ranging from 4 to 8 places (Ialomița, Dâmbovița, Prahova, Bra'ov, Argeș, Buzău, Olt, Vrancea, Dolj, Mureș, Alba, Giurgiu, Hunedoara, Vâlcea, Boto'ani, Bacău, Vaslui, Bihor, Satu Mare, and Caraș‐Severin).

**Figure 14:**
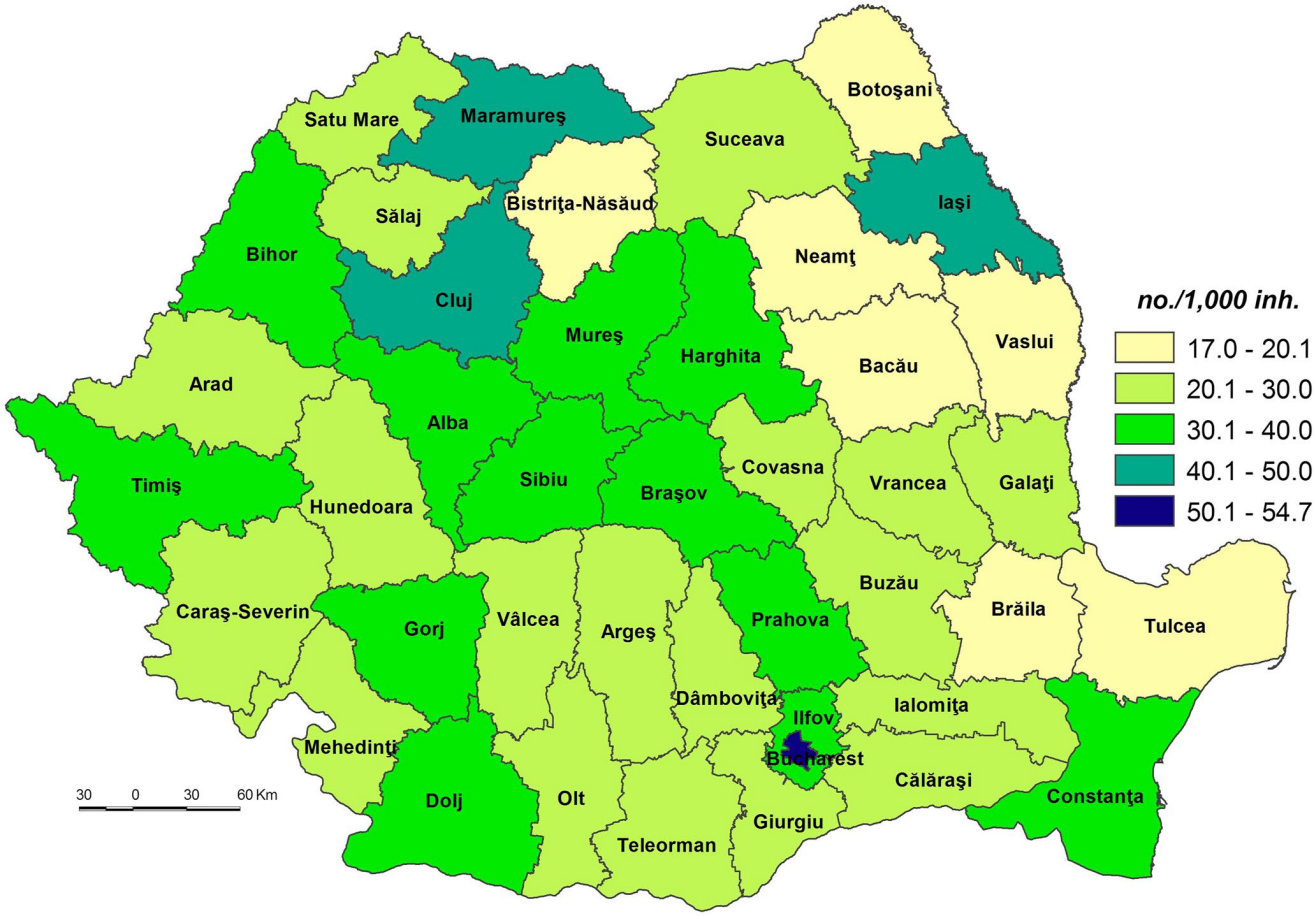
Number of vaccinated persons.

The territorial distribution of the vaccinated population shows that the north‐eastern and eastern counties registered the lowest values caused by the few available vaccination locations (e.g., only 5 in Bacău and Vaslui counties each, and 4 in Tulcea and Brăila counties each). The vast majority of counties (31) fall into the medium classes and 3 counties (Cluj, Maramureș, and Ia'i), together with Bucharest, recorded ones of the highest values (between 40 and 50 persons vaccinated/1,000 inh.) and, respectively, the maximum value (54.7) (Figure 14).

The adaptive capacity is dependent on the way in which the vaccination process was set up and is managed. These facts decrease the efficiency and the purpose of the vaccination process as the main tool to fight the virus. A detailed study focusing on the territorial features of the vaccination process (Mocanu et al., 2021) revealed that it is impacted by several dysfunctions, such as: (a) the concentration in urban settlements (83.2% of total of 357 vaccination centres functioning at the end of February 2021, which means that a large number of people living in large rural areas remain outside vaccination process); (b) the imbalance with the high share of elderly people, making up over 30% of the population (which are registered in the rural settlements from the southern half of Romania, as well as in the western parts, especially in the mountain areas (Gheţău et al., 2016; Mitrică et al., 2019; Mocanu et al., 2021)); (c) the access to the national and county road network. Given these issues, the importance of prevention is crucial and it starts with each individual. Thus, the education level, the access to an efficient healthcare system and to the vital urban services (i.e., water supply network) are only some of the elements which should be considered as strengthening the adaptive capacity.

**Figure 15:**
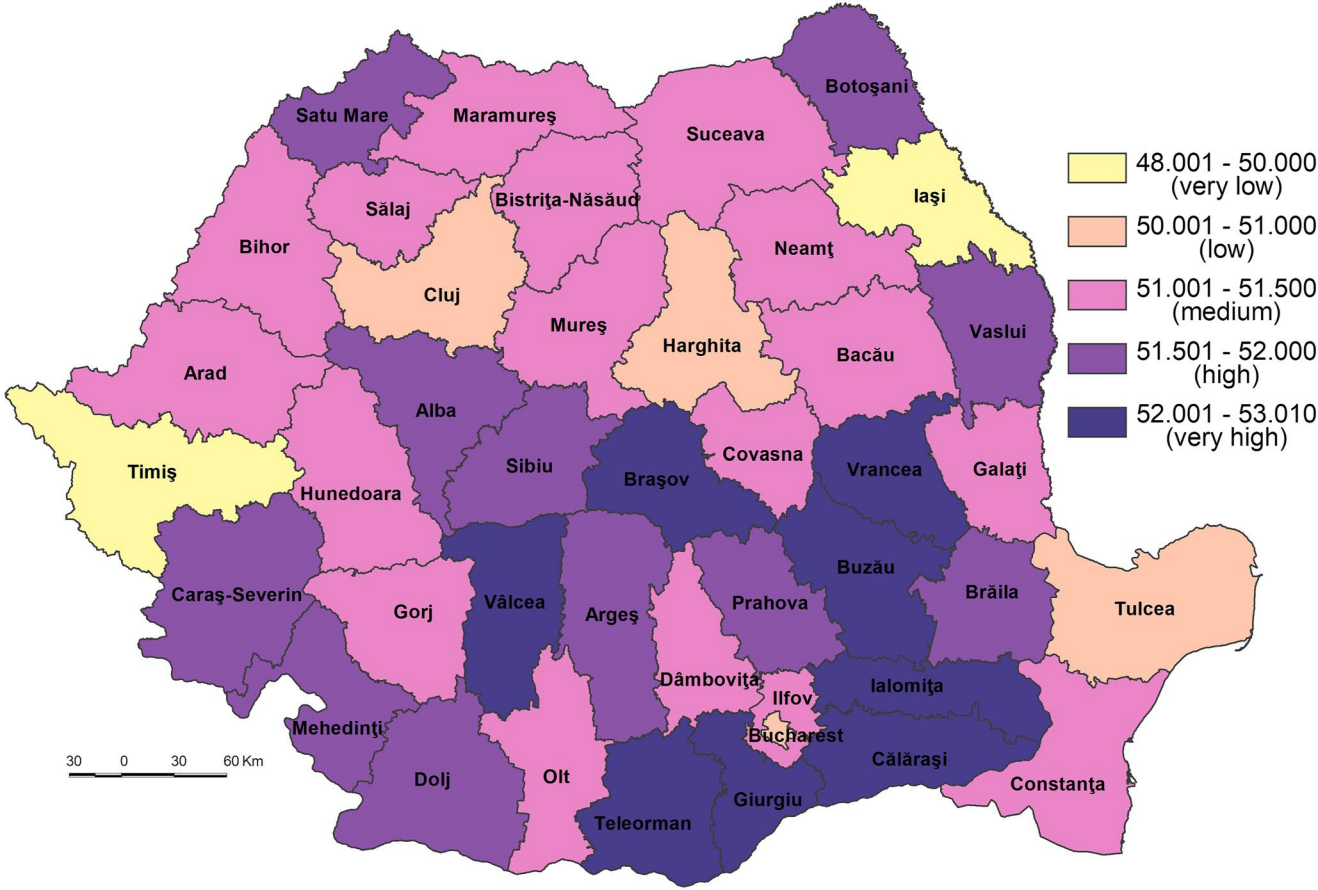
Population Intrinsic Vulnerability to the SARS‐CoV‐2 virus infection Index (PIV_SARS‐CoV‐2).

The **Population Intrinsic Vulnerability to the SARS‐CoV‐2 virus infection Index (PIV_SARS‐CoV‐2)** varies from a maximum of 52.65 in Ialomiţa County to a minimum of 47.52 in Bucharest Municipality (Figure 15) and depends on exposure, sensitivity, coping capacity and adaptive capacity. That means that high vulnerability is correlated with high exposure, and sensitivity with low coping capacity and adaptive capacity.

The very high vulnerability (52.001–53.010) includes nine counties located, mainly, in the south and east of Romania, counties with a low level of social development (Ialomița, Călăra'i, Buzău, Vrancea, Teleorman, Giurgiu, Vâlcea, Bra'ov (Mitrică et al., 2020)). The highest values are attributed to Ialomiţa and Călăraşi Counties, especially as they pertain to the lack of CC_SARS_CoV‐2 and AC_SARS_CoV‐2 and very high sensitivity, even if the exposure is lower. The exception is Bra'ov County in the central part of Romania with a high S_SARS_CoV‐2.

The high vulnerability (51.501–52.000) incorporates 11 counties (Satu Mare, Brăila, Mehedinți, Dolj, Alba, Cara'‐Severin, Boto'ani, Sibiu, Prahova, Vaslui and Argeș) from the south‐west, south, central and north‐east parts of Romania most part of them having also a low level of social development. The exceptions are the Sibiu and Prahova Counties with a high level of socio‐economic development but with high PE_SARS_CoV‐2 and very low CC_SARS_CoV‐2 for Prahova County and low CC_SARS_CoV‐2 for Sibiu County. The difference compared to the previous class stems from the medium to high sensitivity and the high to medium lack of CC_SARS_CoV‐2.

The majority of counties (17) are included in the medium vulnerability class (51.001–51.500) (Figure 15). They are located in different parts of Romania but have the same particularity, namely a medium to high socio‐economic development level, for example, counties from the southern and south‐western (Dâmbovița, Ilfov, Gorj, Olt, and Constanța), central (Covasna and Mureș), north‐east (Suceava, Neamț, Bacău, and Galați), north‐west (Bistrița‐Năsăud, Sălaj, and Maramureș) and western (Hunedoara, Arad and Bihor) parts of the country. In some cases, the PE_SARS_CoV‐2 is medium and is generally characterized by an intense lack of CC_SARS_CoV‐2.

Some of the most well‐developed counties (Bucure'ti and Cluj) registered a low vulnerability level (50.001–51.000). The medium or high exposure of these counties is balanced by a low sensitivity and high adaptive capacity and coping capacity. The other two counties Harghita and Tulcea are characterized by a low exposure and high adaptive capacity.

**Figure 16:**
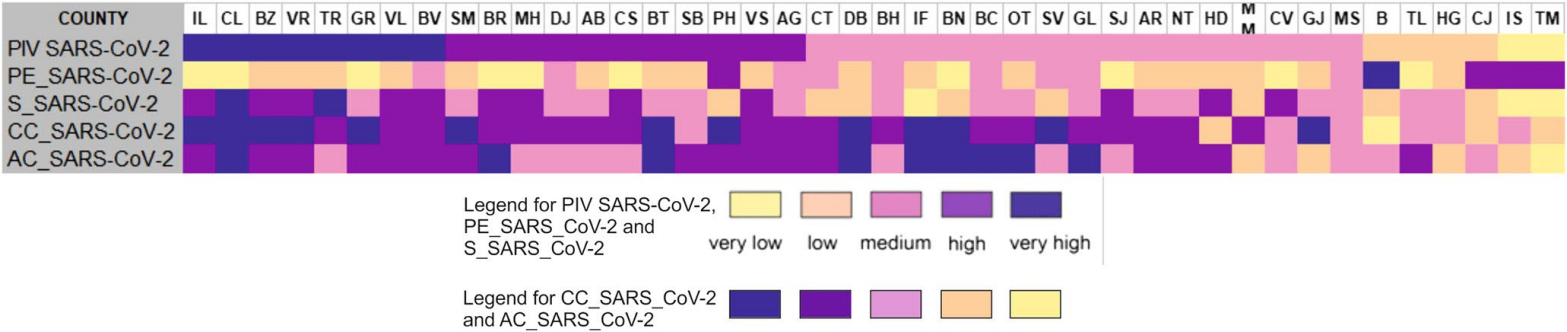
Population Intrinsic Vulnerability to SARS‐CoV‐2 virus infection Index (PIV_SARS‐CoV‐2) by counties and secondary indexes.

Ia'i and Timiş Counties have the lowest vulnerability (48.001–50.000), generally due to the low sensitivity and high adaptive capacity, even the exposure being at ones of the highest level (Figure 16).

## 5 Discussions and Conclusions

Addressing geomedical phenomena spatially in close connection with the key underlying factors, analyzing the location of medical/emergency services and the response capacity of the healthcare system in crisis situations, identifying vulnerable groups at different scales/territorial levels are all essential aspects to be considered when analyzing health‐related issues from a geographical perspective. Thus, in addition to the medical and epidemiological research, geographic vulnerability analysis plays an essential role in understanding the characteristics of an outbreak (i.e., the SARS‐CoV‐2 virus infection) in relation to a number of underlying factors (intrinsic and extrinsic).

Although the international concerns with respect to the SARS‐CoV‐2 virus have been and are still generally related to medical and epidemiological issues, impact on health, the dynamics of the spread in relation to a number of determinants (intrinsic and extrinsic) etc., understanding the socio‐demographic preconditions (e.g., age, sex, and occupation) are vital elements that can help quantify the pandemic potential and anticipate the likely number of deaths (Jung et al., 2020; Kucharski et al., 2020) in order to take the necessary measures in order to limit the spread.

The mapping of the different degrees of vulnerability could solve the visibility problem of possible areas with a vulnerable population, as well as the communication problem between different institutional health levels (e.g., regional, county, city hospitals, clinics, medical offices) and administrative ones (Counties), but also between all of them and the local communities (cities, rural communes) and/or professionals (medical staff, pharmacists). According to the vulnerability level, the territory of Romania is split in two parts: (a) the southern and north‐eastern counties mainly defined by a very high and high degree of vulnerability. The exceptions are Iaşi and Constanţa Counties featuring a low degree of vulnerability due to a good performance in terms of CC_SARS_CoV‐2 and AC_SARS_CoV‐2; (b) the central and western counties marked by a medium and very low vulnerability due to the very low sensitivity factor; Cluj, Timiş and Hunedoara Counties are, after Bucharest Municipality, the less vulnerable.

At the national level, the correlation between rank (by confirmed cases of SARS‐CoV‐2 virus infection) and the population’s vulnerability to the SARS‐CoV‐2 virus infection point a medium inverse relationship (Pearson = −0.254) but with no statistically significance (*p*‐value = 0.130). The Pearson correlation between each component indicator of the PIV_SARS‐CoV‐2 and confirmed cases of SARS‐CoV‐2 virus infection, indicate an inverse relationship in the case of dwellings not connected to the public water network supply (Pearson = −0.681; *p* value = 0.000), PCR testing laboratories (Pearson = −0.586; *p* value = 0.000), vaccinated persons (Pearson = −0.572; *p* value = 0.000), Assistance and Intensive Care bed capacity (Pearson = −0.506; *p* value = 0.001), available places in vaccination locations (Pearson = −0.483; *p* value = 0.001).

**Figure 17:**
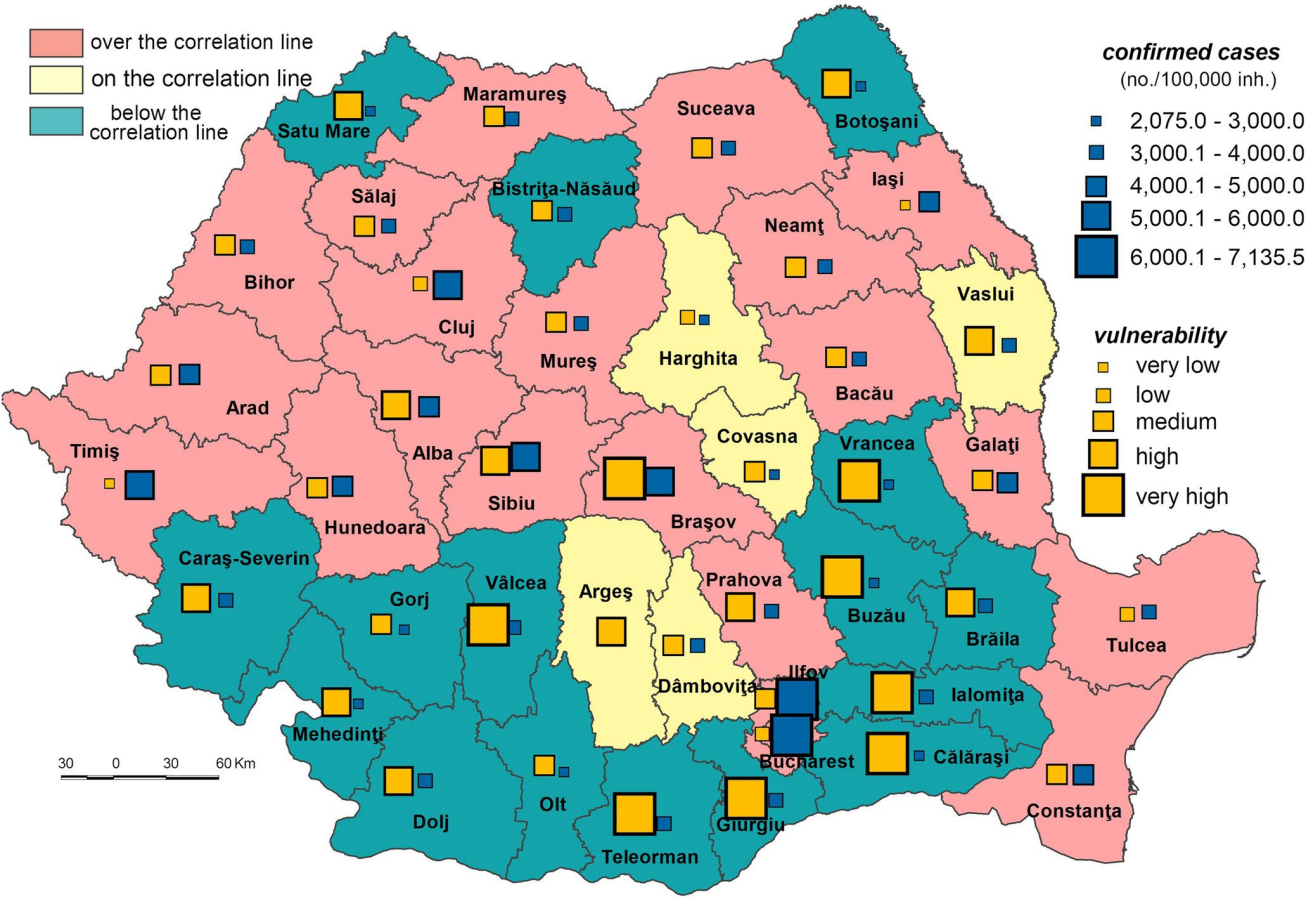
The correlation between the vulnerability to the SARS‐CoV‐2 virus infection and the number of confirmed cases.

The analysis of counties according to their categories (i.e., influenced by their position relative to the correlation line: on, below or over) reveals several important aspects in terms of not only confirmed cases of SARS‐CoV‐2 virus infection, but of other elements somehow connected to the situation of infections produced by this virus. The detailed analysis of the statistical correlation shows that (Figure 17):

1. A number of five counties (Harghita, Covasna, Vaslui, Dâmbovița, and Argeș), scattered throughout the country, are positioned on the correlation line, meaning that there is a relationship between the vulnerability level and confirmed cases.
2. The 16 counties positioned below this line registered a poor rank/size correlation. They are territorially grouped in the north‐east (Vrancea and Boto'ani), and south (Buzău, Brăila, Ialomiţa, Călăraşi, Giurgiu, and Teleorman) and south‐west (Olt, Vâlcea, Gorj, Dolj, Mehedinţi, and Cara'‐Severin counties) and north‐west (Bistrița‐Năsăud and Satu Mare). However, the position of some of these counties (i.e., Giurgiu and Dolj) should be linked to the indicators showing the last places occupied in the hierarchy of the PCR testing laboratories/100,000 inh. and the AIC beds/1,000 inh and first place in the case of Teleorman County for number of people suffering from cardiovascular diseases. According to previous explanations, this could be a positive fact for these counties which are marked by a lower level of confirmed cases than the vulnerability level.
3. Counties located in the central, western and south‐eastern part of Romania are positioned over the correlation line, meaning that the number of SARS‐CoV‐2 confirmed cases is higher compared to population vulnerability. The explanation consists of the fact that large exposure and low sensitivity are very important. Some counties are added to this location group, namely Iaşi, Galaţi, Constanţa, Ilfov, and Prahova, which display a high exposure because of the existence of metropolitan areas. Thus, a high number of confirmed cases could be a reflection of various facts: a relatively great impact of the exposure intensity or a high level of awareness of the population regarding the importance of complying to the rules of behaving in private and public spaces. The reverse situation is also valid.

The results of such a methodological approach could lead to a rethinking of the interventions of the decision‐makers in the present case, but also in the case of any future pandemics. It can help reduce the economic impact by more efficiently involving local/regional actors in identifying targeted solutions, in contrast to generalized ones. It can also contribute to a better understanding and improvement of social cohesion through measures aimed at mitigating the vulnerability of the population and helping decision‐makers to better anticipate and respond to social challenges, as well as contribute to shaping national/regional development strategies and practices that respond to social challenges, and improve health infrastructure strategies (physicians and medical staff, health facilities) in disadvantaged regions based on lessons learnt from other regions in Europe and good practice examples. Thus, the role of such a study may be to prepare stakeholders to improve local governance by building responses at national, regional and local levels more tailored to the particular needs of each county, city or commune.

## Conflict of Interest

The authors declare no conflicts of interest relevant to this study.

## Data Availability

The data used in the present study were derived from the following resources available in the public domain: National Institute of Statistics at http://statistici.insse.ro:8077/tempo-online/#/pages/tables/insse-table; in Romanian Government at https://vaccinare-covid.gov.ro/platforma-programare/ (Covid vaccination platform programming), only available in Romanian; Coronavirus COVID‐19 Romania at https://covid19.geo-spatial.org/harti/hospital-infrastructure; Ministry of Health at https://data.gov.ro/dataset/transparenta-covid; Covid‐19 Știri Oficiale (Covid‐19 Official news) at https://stirioficiale.ro, only available in Romanian; Covid‐19 Date la zi (Covid‐19 Updated) at https://datelazi.ro/, only available in Romanian.
